# Vitamin D and Neonatal Sepsis: Current Evidence, Biological Mechanisms, and Future Directions—A Scoping Review

**DOI:** 10.3390/nu18152517

**Published:** 2026-08-03

**Authors:** Dominika Paw, Renata Bokiniec, Krzysztof Włodarczyk, Alicja Kołodziejczyk-Nowotarska

**Affiliations:** 1 Department of Neonatology and Neonatal Intensive Care, Medical University of Warsaw, 00-315 Warsaw, Poland; 2 Main Library, Medical University of Warsaw, 02-091 Warsaw, Poland

**Keywords:** infant, neonatal sepsis, vitamin D deficiency, innate immunity, 25-hydroxyvitamin D, vitamin D receptor

## Abstract

**Objective:** Neonatal sepsis is a major cause of morbidity and mortality. Vitamin D modulates immune responses and influences the risk and outcomes of neonatal sepsis. Therefore, we aimed to systematically outline the role of vitamin D in neonatal sepsis, including its effects on incidence, severity, treatment outcomes, and underlying mechanisms. **Methods:** A literature search was conducted in the MEDLINE and EMBASE databases with English language restrictions and no date limit. Eligible sources included experimental, observational, and descriptive studies as well as reviews addressing vitamin D status, supplementation, and mechanisms in neonatal sepsis. This review followed the PRISMA Extension for Scoping Reviews guidelines. **Results:** Forty-five studies were selected and analyzed. Vitamin D deficiency was globally prominent among neonates and may be correlated with an increased risk or severity of sepsis. Most studies were observational, primarily case-control or cross-sectional, and conducted in South Asia and the Middle East, with increasing research activity after 2018. The number of term infants was higher than that of preterm infants. Recently, innate immune mechanisms, including antimicrobial peptides and toll-like receptor signaling, were highlighted. However, comparisons across studies were limited by inconsistencies in design, vitamin D cut-offs, timing of blood sampling, and sepsis definitions. Only six studies included patients with culture-proven sepsis, whereas others included clinically suspected cases. Few studies assessed the long-term outcomes, immune maturation, or neurodevelopmental consequences. **Conclusions:** Vitamin D deficiency may be associated with neonatal sepsis; however, the findings remain inconsistent because of methodological variability. Therefore, high-quality, standardized, multicenter studies are needed. The review also identified substantial methodological heterogeneity regarding vitamin D assessment, sepsis definitions, and neonatal populations, which currently limits evidence-based clinical recommendations.

## 1. Introduction

Neonatal sepsis is a major cause of neonatal morbidity and mortality worldwide. Neonatal sepsis is a systemic inflammatory response syndrome associated with a proven or suspected infection. It is particularly prevalent among extremely low birth weight (ELBW, <1000 g) neonates at significantly higher risk due to their immunological immaturity and frequent reliance on invasive medical interventions, such as mechanical ventilation, central venous catheterization, and parenteral nutrition [1]. Both early-onset sepsis (EOS), occurring within the first 72 h of life, and late-onset sepsis (LOS), which occurs after 72 h of life, are critical challenges in neonatal care [2]. This constitutes a substantial public health challenge for low- and middle-income countries (LMICs). According to a meta-analysis, the incidence of neonatal sepsis is substantially higher in LMICs than in high-income countries. The highest burden was observed in Africa and South-East Asia. EOS occurred approximately 2.6-fold more frequently than LOS, particularly among preterm and very low birthweight neonates, whereas the overall mortality associated with neonatal sepsis was 17.6%, underscoring the severe clinical and epidemiological impact of this condition in resource-limited settings [3].

The impact of neonatal sepsis extends far beyond the acute phase; survivors are at a heightened risk of long-term neurodevelopmental impairments, including cerebral palsy, cognitive deficits, and visual or hearing loss. Recurrent or persistent episodes of sepsis are associated with increased mortality, prolonged hospital stays, and substantial healthcare costs [4]. Therefore, novel biomarkers and therapeutic strategies to mitigate the risk of sepsis and improve neonatal outcomes are required.

Vitamin D is a crucial immunomodulatory molecule with the potential to influence neonatal susceptibility to infections, such as sepsis. Traditionally recognized for its role in calcium and bone metabolism, vitamin D plays a key role in regulating the immune system. The active form of vitamin D, 1,25-dihydroxyvitamin D, exerts its effects through the vitamin D receptor (VDR), a transcription factor that modulates the expression of genes involved in immune function [5]. Notably, vitamin D enhances the production of antimicrobial peptides, including cathelicidin and β-defensins, which are first-line defense mechanisms against several pathogens. Additionally, vitamin D influences the differentiation and activation of various immune cell types, including macrophages, dendritic cells, and T lymphocytes, thereby shaping both innate and adaptive immune responses [6,7].

Despite its critical role in immune regulation, vitamin D deficiency is concerning. The maternal vitamin D status directly affects neonatal vitamin D reserves; maternal vitamin D deficiency leads to inadequate vitamin D stores in infants [8]. Vitamin D deficiency occurred in more than half of pregnant women and newborns in five global regions [8]. This is particularly problematic in ELBW neonates, who are more likely to exhibit severe vitamin D deficiency at birth due to limited placental transfer and insufficient sun exposure [9]. Furthermore, preterm infants hospitalized in NICU settings are particularly vulnerable to vitamin D deficiency owing to limited stores, prolonged hospitalization, and delayed establishment of enteral supplementation, which may contribute to increased susceptibility to LOS and immune dysfunction [10,11].

Vitamin D may play a role in the neonatal sepsis risk. A meta-analysis of observational studies demonstrated a significant association between low serum vitamin D levels and an increased incidence of sepsis in critically ill children, including neonates [12]. Genetic variations in the *VDR* gene have been implicated in susceptibility to infectious diseases. Polymorphisms, such as FokI, BsmI, TaqI, and ApaI, may influence VDR function and modulate host immune responses to bacterial and viral pathogens [13]. A recent study confirmed the relationship between VDR polymorphisms and increased sepsis risk in neonates, suggesting that genetic factors may interact with environmental variables, such as vitamin D status, to influence infection outcomes [14].

Current study findings on the relationship between vitamin D status and neonatal sepsis are inconsistent. The studies considerably vary with respect to vitamin D assessment methods, population characteristics, definitions, diagnostic criteria for sepsis, and outcome reporting. This variability limits the establishment of robust conclusions. A scoping review enables the systematic mapping of key concepts, identification of knowledge gaps, and characterization of methodological approaches across diverse studies.

Emerging evidence continues to support a potential association between vitamin D status and neonatal sepsis. López-Garrido et al. reported that lower serum vitamin D concentrations at birth were associated with an increased risk of late-onset sepsis in preterm infants [15]. Furthermore, the umbrella review by Castro-Luna et al. concluded that, although low vitamin D levels are consistently associated with adverse sepsis outcomes, substantial methodological heterogeneity continues to preclude definitive clinical recommendations [16]. Collectively, these findings further emphasize the need for comprehensive evidence mapping and well-designed prospective studies.

In this scoping review, we examine the current state of vitamin D supplementation practices in NICU settings, with a particular focus on the challenges associated with delayed supplementation in ill infants and the potential benefits of early intervention. We aim to systematically outline the relationship between neonatal vitamin D levels and the risk of sepsis using available information.

## 2. Materials and Methods

This review was conducted in accordance with the methodological framework originally proposed by Arksey and O’Malley and subsequently refined by the Joanna Briggs Institute [17,18]. Furthermore, the reporting of this review followed the Preferred Reporting Items for Systematic Reviews and Meta-Analyses Extension for Scoping Reviews (PRISMA–ScR) guidelines to ensure transparency, reproducibility, and methodological rigor [19].

We selected a scoping review methodology to map the available evidence, characterize the current state of knowledge, and identify gaps in the literature. Given the broad and evolving nature of the research topic, a scoping approach allowed for the inclusion of diverse study designs and facilitated the exploration of key concepts, research trends, and areas requiring further investigation. The review protocol was prospectively developed and registered in the Open Science Framework (OSF; https://doi.org/10.17605/OSF.IO/69ZK7) before the literature search and study selection commenced. Prospective registration enhanced the transparency of the review process, improved methodological reproducibility, and minimized the risk of reporting bias.

The following research questions were used to guide the review:

1. What research exists on vitamin D status or supplementation in relation to sepsis in neonates?
2. What is known about the relationship between maternal and neonatal vitamin D levels or supplementation and the occurrence of EOS or LOS in neonates?
3. What biological mechanisms have been proposed to explain the relationship between vitamin D levels and neonatal immune function and susceptibility to sepsis?
4. What differences in vitamin D status, immune responses, and sepsis risk have been reported between preterm and term neonates?
5. What progress has been made in the study of vitamin D levels and neonatal sepsis over time?
6. What are the gaps, limitations, or underexplored areas in the current literature regarding vitamin D and sepsis in the neonatal population?

A completed PRISMA-ScR checklist is provided as Supplementary File S1.

### 2.1. Literature Search and Selection

An initial exploratory search was conducted to familiarize the authors with the scope and characteristics of the existing literature, identify relevant terminology, and develop a comprehensive search strategy. Based on this preliminary assessment, search terms and information sources were selected using established keywords and controlled vocabulary (e.g., MeSH and Emtree terms) commonly applied in research on vitamin D and neonatal outcomes. The final search strategy was reviewed, refined, and executed in collaboration with an experienced research librarian to ensure completeness and methodological rigor.

A literature search was performed using two electronic databases: MEDLINE (via PubMed) and EMBASE. No publication date restrictions were applied; however, the search included studies published up to October 2025 (Appendix A). Gray literature and unpublished studies were excluded. Additionally, records from ClinicalTrials.gov were screened, along with peer-reviewed publications, to identify ongoing or recently completed studies. The registry was searched using combinations of the following keywords: “vitamin D”, “vitamin D deficiency”, “cholecalciferol”, “neonate”, “newborn”, “infant”, and “sepsis”. Trials without available results were retained for descriptive mapping purposes only, whereas records that did not meet the inclusion criteria or had a withdrawal or termination status were excluded.

Following the removal of duplicate records using EndNote, all the remaining citations were imported into Covidence (https://www.covidence.org/) for screening [20,21]. Two reviewers independently screened the titles and abstracts. Eligibility was limited to studies involving preterm or term neonates aged ≤28 days. Irrelevant studies were excluded from the analysis. Given that abstracts frequently lack detailed information on participant characteristics, studies were excluded at this stage only if they explicitly indicated a non-neonatal population or an older age group. Full-text articles were retrieved from all potentially relevant records and independently assessed by two reviewers using predefined inclusion and exclusion criteria (Table 1). Disagreements between reviewers were resolved through discussion and, when necessary, consultation with a third reviewer. The primary reason for exclusions was recorded for each full-text article (Appendix B), in accordance with PRISMA-ScR recommendations to ensure transparency and reproducibility of the study selection process.

### 2.2. Data Extraction

Data were charted using a pre-established data extraction form developed within Covidence and structured in accordance with the population-conceptual-context framework recommended for scoping reviews. The extraction form captured key study characteristics (e.g., study design, setting, and population), details on vitamin D exposure (including measurement methods and cut-off values), neonatal sepsis outcomes (definitions and diagnostic criteria), reported biological mechanisms, emerging research trends, and knowledge gaps.

The data-charting form was iteratively refined throughout the review process to ensure completeness, clarity, and consistency of data capture across studies. Data extraction was independently performed by two reviewers using a standardized approach, with discrepancies resolved through discussion and, where necessary, consultation with a third reviewer.

To avoid evidence duplication, primary studies included in the two eligible systematic reviews and meta-analyses were not re-extracted. Instead, data from these sources were charted only at the review level, consistent with the objective of mapping the existing evidence base rather than synthesizing individual study outcomes.

Consistent with the methodological purpose of a scoping review, no formal risk of bias or methodological quality assessments were conducted. The extracted data were descriptively presented by summarizing the study characteristics and mapping the reported associations between vitamin D status and neonatal sepsis, as well as highlighting patterns, inconsistencies, and gaps in the literature.

## 3. Results

The included studies differed substantially with respect to study design, study population, vitamin D assessment, and sepsis definitions. Therefore, the findings are presented according to major study characteristics to facilitate comparison across studies.

### 3.1. Selection of Sources of Evidence

After the selected records were imported into the reference management software Covidence and duplicates were removed, 457 records were identified. After de-duplication and title and abstract screening, 384 records remained [21]. Sixty full-text articles were retrieved and assessed for eligibility. Of them, 45 met the predefined inclusion criteria and were included in the scoping review.

For records that appeared potentially eligible based on title and abstract screening but whose full texts could not be retrieved, additional searches were performed using institutional library subscriptions, publisher websites, PubMed, Google Scholar and interlibrary search services. Records for which the complete manuscript remained unavailable despite these efforts were retained in the PRISMA flow diagram and listed in Appendix B as “No article found” to ensure transparency of the study selection process.

**Figure 1:**
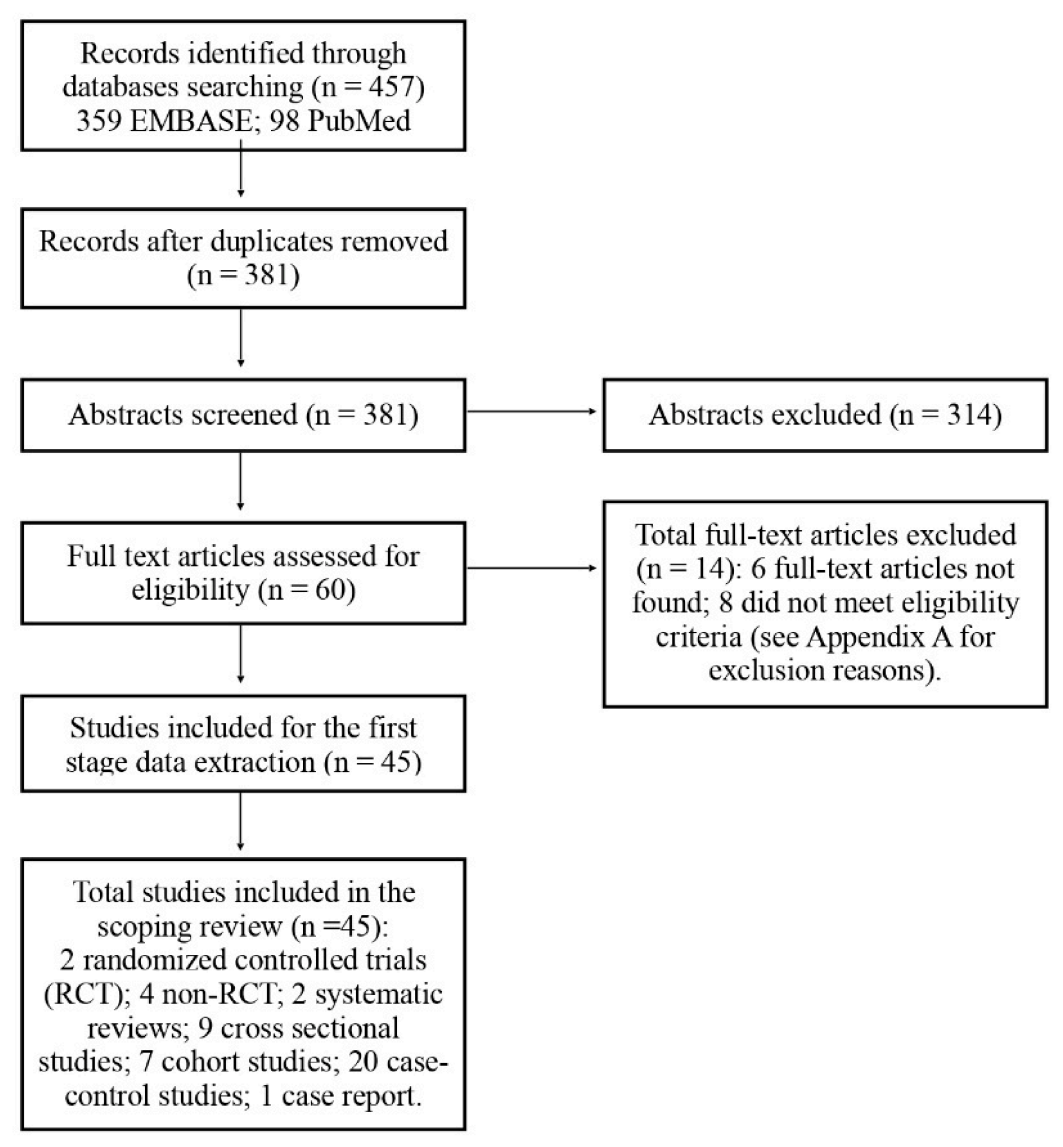
Literature search and study selection process.

All the included studies involved neonatal populations and examination of the association between vitamin D status and neonatal sepsis or sepsis-related outcomes. The evidence primarily consisted of original research articles and two systematic reviews. The study selection process is presented in Figure 1 in accordance with PRISMA guidelines.

### 3.2. Characteristics of Sources of Evidence

**Figure 2:**
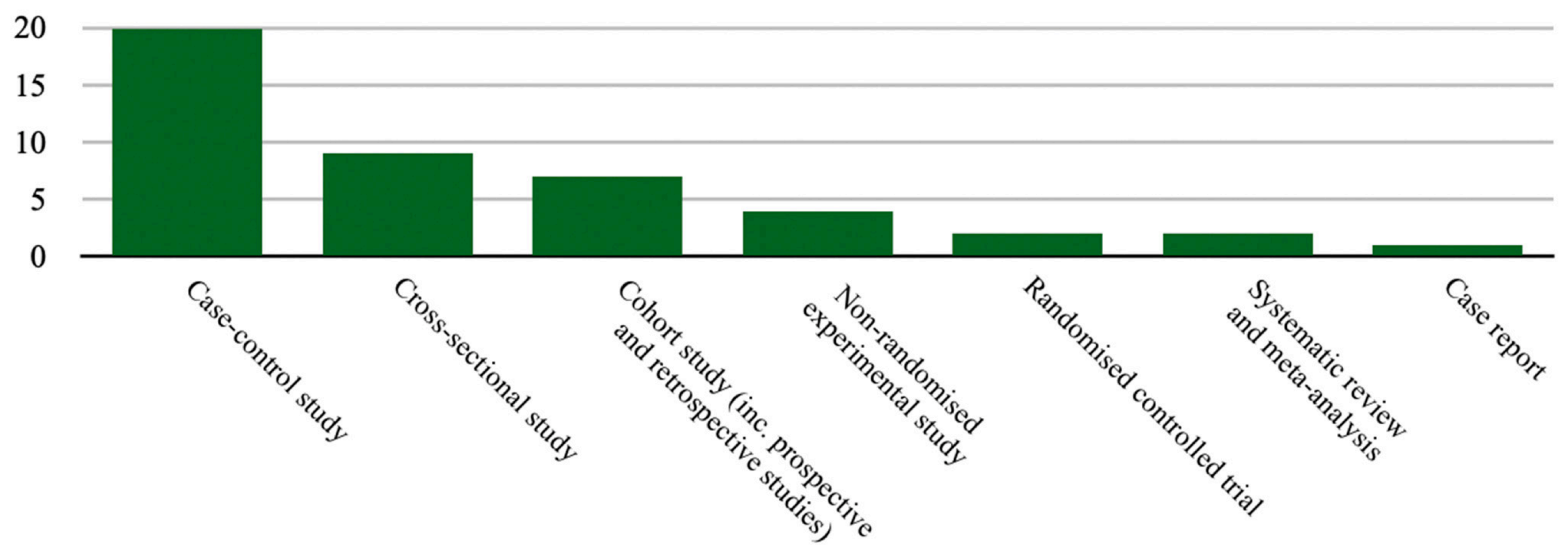
Distribution of the included studies by study design.

The included studies were predominantly observational. Case-control studies constituted the largest proportion (n = 20, 44.4%), followed by cross-sectional studies (n = 9, 20.0%); cohort studies, including prospective and retrospective designs (n = 7, 15.6%); non-randomized experimental studies (n = 4, 8.9%); and randomized controlled trials and systematic reviews with meta-analyses (n = 2 each, 4.4% each). Case reports were the least frequently used study design, with only one study (2.2%) included. Overall, the distribution of the study designs indicates that the current literature is largely based on analytical observational approaches, with relatively few randomized investigations (Figure 2).

**Figure 3:**
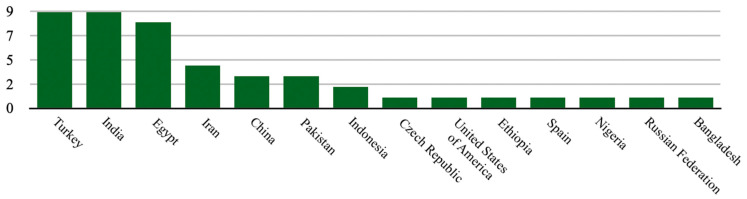
Geographic distribution of the included studies by country.

Geographically, studies were predominantly conducted in India (n = 9, 20.0%) and Turkey (n = 9, 20.0%), followed by Egypt (n = 7, 15.6%), Iran (n = 4, 8.9%), Pakistan, and China (n = 3, 6.7%). Single studies were conducted in Indonesia, the Czech Republic, the Russian Federation, the United States, Ethiopia, Spain, Nigeria, and Bangladesh. This distribution indicated a predominance of studies from South Asia and the Middle East, with limited representation from Western Europe and North America (Figure 3).

**Figure 4:**
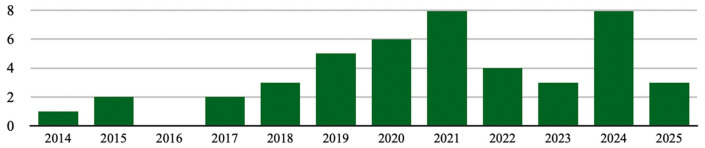
Distribution of the included studies by publication year.

The highest research activity was observed in 2021 and 2024 (n = 8 each; 17.8%). A significant increase was also observed in 2020 (n = 6; 13.3%) and 2019 (n = 5; 11.1%). Overall, the data demonstrate a marked upward trend in research output after 2018, suggesting a recent increase in scientific interest in the topic (Figure 4). A detailed description of the selected studies is provided in Supplementary File S2.

Most studies were conducted in tertiary-care or university-affiliated hospital settings and included neonates diagnosed with sepsis or evaluated for sepsis risk, as well as healthy control groups [22,23,24,25,26,27,28,29,30,31,32,33,34,35,36,37,38,39,40,41,42,43,44,45,46,47,48,49,50,51,52,53,54,55,56,57,58,59,60,61,62,63,64,65,66,67]. Both term and preterm neonates were reported, although many studies focused primarily on term infants [24,25,26,27,29,31,34,38,43,44,50]. The outcomes investigated included EOS, LOS, culture-confirmed sepsis, and clinically diagnosed sepsis. The sample sizes substantially varied between studies, ranging from one case report to small single-center cohorts to larger observational populations [22,24,25,26,27,28,29,30,31,32,33,34,35,36,37,38,39,40,41,42,43,44,45,46,47,48,49,50,51,52,53,54,55,56,57,58,59,60,61,62,63,64,65,66,67]. Only six investigations included culture-proven sepsis cases, whereas others included neonates with clinically suspected sepsis, contributing to methodological heterogeneity [25,28,33,39,46,51].

Several studies demonstrated that severe vitamin D deficiency is more frequent among neonates with confirmed sepsis and is associated with less favorable clinical outcomes, including positive blood cultures, septic shock, increased inflammatory response, and high mortality [28,36,47,60,62]. Associations between lower vitamin D concentrations and disease severity indicators, including immune dysregulation, elevated inflammatory markers, prolonged hospitalization, and worse clinical course, have also been reported [28,34,37,43,47,53,67]. However, these outcomes were not consistently assessed across all included studies [48].

Considerable heterogeneity was observed in the definitions of vitamin D deficiency, with thresholds ranging from <10 ng/mL to <30 ng/mL across studies [22,24,25,26,27,28,29,30,31,32,33,34,35,36,37,38,39,40,41,42,43,44,45,46,47,48,49,50,51,52,53,54,55,56,57,58,59,60,61,62,63,64,65,66,67]. Interventional approaches were evaluated less frequently than observational associations, and only a limited number of studies assessed maternal or neonatal vitamin D supplementation [28,31,32,37,38,39,41,48,52,54,58,63,67].

### 3.3. Maternal and Neonatal Vitamin D Status

A key theme emerging from the included studies was the strong relationship between maternal and neonatal vitamin D status. Several studies suggested that maternal vitamin D deficiency may contribute directly to an impaired neonatal vitamin D status, as positive correlations between maternal and neonatal 25(OH)D concentrations were consistently observed [27,30,38,66]. Most of the included studies reported low maternal, cord blood, or neonatal vitamin D concentrations associated with neonates with sepsis compared to those of healthy controls [24,25,28,29,38,43,44,50,59,60,62,65,66]. Maternal vitamin D supplementation during pregnancy was also associated with a low EOS risk [31].

Associations between vitamin D deficiency and EOS and LOS were described in multiple studies [24,25,29,30,33,45,54,61]. Vitamin D concentrations <20 ng/mL are frequently observed in preterm infants who developed sepsis [54]. Low vitamin D concentrations were also associated with greater disease severity, septic shock, impaired immune markers, and high mortality [28,36,47]. Overall, studies investigating EOS generally demonstrated more consistent associations between lower vitamin D concentrations and sepsis risk than studies evaluating LOS. Nevertheless, findings for both EOS and LOS remained heterogeneous owing to differences in study design, diagnostic criteria, and adjustment for confounding variables.

However, the results were not entirely consistent. Some studies did not identify a significant association between vitamin D concentration and neonatal sepsis [26,39,46,49,52], whereas others reported mixed or inconclusive results [51,56]. One review concluded that available evidence was insufficient to establish a causal relationship [48]. Recent studies expanded the analysis beyond total 25(OH)D concentrations by evaluating vitamin D metabolites and vitamin D receptor polymorphisms that are potentially associated with neonatal susceptibility to infection [57,64].

### 3.4. Biological Mechanisms Linking Vitamin D and Neonatal Immunity

The included literature provided increasing insight into the biological plausibility underlying the association between vitamin D deficiency and neonatal sepsis. Vitamin D was consistently implicated in the modulation of innate immune responses, particularly through its role in inducing antimicrobial peptides, such as cathelicidin and β-defensins, which are critical components of the host defense [68]. This mechanism was described in multiple studies that evaluated neonatal innate immunity and antimicrobial peptide regulation [24,25,26,30,32,37,38,45,50,53,55,58,60,61,62,65,66,67]. Additionally, several studies demonstrated that vitamin D influences cytokine regulation by suppressing pro-inflammatory mediators, including interleukin-6 and tumor necrosis factor-alpha, and promoting anti-inflammatory pathways [25,29,31,47,49,67]. This immunomodulatory effect was particularly relevant in neonates whose immune systems are inherently immature and prone to dysregulated inflammatory responses. Additional biological mechanisms proposed across studies included TLR signaling modulation, neutrophil and macrophage function enhancement, epithelial barrier integrity maintenance, and autophagy pathway regulation [27,39,44,55,60]. Associations between low vitamin D levels and elevated inflammatory biomarkers, such as C-reactive protein and procalcitonin, were reported in multiple studies, further supporting the link between vitamin D deficiency and exaggerated inflammatory responses during sepsis. Elevated vitamin D levels during acute sepsis were also reported, suggesting that dynamic alterations in serum 25(OH)D levels may reflect the activation of inflammatory and immune pathways during infection [22]. Interventional evidence remains limited but suggests potential therapeutic effects. Vitamin D supplementation was associated with improved sepsis scores and reduced levels of inflammatory markers in neonates [37].

### 3.5. Differences Between Preterm and Term Neonates

Many studies included both term and preterm neonates, although direct comparisons between these groups were relatively uncommon [24,27,30,36,38,43,44,45,53,54,60]. Preterm infants consistently exhibited lower vitamin D concentrations than those of term neonates, likely due to reduced placental transfer during the third trimester, lower body stores, and limited postnatal intake [24,30,38,54]. Premature birth was also an independent risk factor for neonatal sepsis, particularly EOS and LOS [24,45,54,61,63]. Several studies have suggested that premature birth and vitamin D deficiency may have additive effects on infection susceptibility [30,36,53,54,60]. Preterm infants with vitamin D concentrations <20 ng/mL showed high rates of LOS and inflammatory complications [54]. Low gestational age was also associated with low vitamin D levels, impaired immune parameters, and increased infection risk [30,34,53,60]. However, most studies did not stratify immune responses or vitamin D metabolism according to gestational age, and detailed subgroup analyses remained limited [34,42,53].

### 3.6. Temporal Trends and Advances in the Field

Earlier studies were predominantly descriptive and focused on the association between vitamin D levels and sepsis incidence [22], whereas more recent studies have incorporated prospective designs, multivariate analyses, and mechanistic investigations. Since 2015, studies introduced more comprehensive analyses of innate immune mechanisms, including antimicrobial peptides and TLR signaling [24,25]. Subsequent investigations have expanded scopes that include adaptive immunity, lymphocyte subsets, placental inflammation, and cytokine regulation [34,35,53,67].

Advances in laboratory methodologies, including more standardized assays for 25(OH)D measurement, improved the comparisons across studies. The 3-epi-25(OH)D3 metabolites and VDR polymorphisms associated with immune regulation were examined in recent studies [57,64]. The interest in vitamin D supplementation increased over time. Maternal supplementation during pregnancy and as an adjunctive intervention for neonatal sepsis was also investigated [31,37]. Despite these advancements, randomized controlled trials remained limited, and the field continues to heavily rely on observational evidence.

### 3.7. Summary of Findings

Lower serum 25(OH)D concentrations in neonates with sepsis than in healthy controls were consistent across studies [24,25,27,29,38,43,44,50,59,60,62,65,66]. The association between vitamin D deficiency and increased risk of neonatal sepsis was reported in both case-control and cohort studies involving both EOS and LOS [24,25,29,30,33,45,54,61]. Low vitamin D concentrations were also linked with disease severity, septic shock, impaired immune markers, prolonged hospitalization, and high mortality in several studies [28,36,47]. Multiple studies also reported relationships between vitamin D status and inflammatory or immunological biomarkers, including CRP, IL-6, TNF-α, white blood cell count, procalcitonin, platelet count, lymphocyte subsets, and antimicrobial peptides, such as LL-37 [25,28,34,37,47,53,58,67]. Inverse associations between serum 25(OH)D concentrations and inflammatory markers were frequently observed, suggesting a potential relationship between vitamin D deficiency and dysregulated inflammatory responses during neonatal sepsis [25,28,37,47,67]. Despite these findings, the magnitude and independence of the reported associations vary among studies [26,39,46,49,51,52,56]. Considerable heterogeneity was observed in study design, vitamin D deficiency thresholds, sepsis definitions, and adjustments for potential confounding factors [22,24,25,26,27,28,29,30,31,32,33,34,35,36,37,38,39,40,41,42,43,44,45,46,47,48,49,50,51,52,53,54,55,56,57,58,59,60,61,62,63,64,65,66,67]. Due to the predominance of observational designs and limited randomized evidence, causality cannot be established based on the current literature.

### 3.8. Gaps Identified

Several important gaps were identified in the literature. Observational case-control or cross-sectional designs and relatively small cohorts (<100 septic neonates) were used in most studies. Interventional evidence remains scarce, with only two randomized controlled trials in the dataset [28,58]. Additionally, supplementation strategies were evaluated in a limited number of studies [28,31,32,37,38,39,41,48,52,54,58,63,67]. Substantial heterogeneity existed in the definition of vitamin D deficiency, laboratory methods, timing of blood sampling, and diagnostic criteria for EOS and LOS. Third, relatively few studies involved long-term neonatal outcomes, immune maturation, and neurodevelopmental consequences of vitamin D deficiency and neonatal infection. Data regarding optimal vitamin D supplementation regimens during pregnancy or the neonatal period were also limited. The geographic concentration of the studies in specific regions may limit the generalizability of the findings to larger populations (Figure 4). Finally, the mechanistic evidence remains incomplete. Although multiple studies proposed pathways involving antimicrobial peptides, cytokine modulation, and VDR signaling, most findings were based on indirect biomarker analyses rather than functional immune assays or longitudinal mechanistic studies. Therefore, standardized diagnostic criteria, harmonized laboratory methodologies, multicenter prospective designs, and adequately powered randomized controlled trials are required to evaluate maternal and neonatal vitamin D interventions.

## 4. Discussion

### 4.1. Summary of Evidence

Interpretation of the available evidence should consider the substantial methodological heterogeneity among the included studies. Differences in study populations included the proportions of term and preterm infants, birth weight categories, geographic regions, and NICU admission criteria. Furthermore, studies used varying definitions of neonatal sepsis, ranging from clinically suspected sepsis to culture-confirmed infection, making direct comparison difficult. Considerable variability also existed in vitamin D assay methodologies, including immunoassays and LC-MS/MS, the thresholds used to define vitamin D deficiency, the timing of maternal, cord blood, or neonatal sample collection, and the extent to which studies adjusted for important maternal and neonatal confounders. Consequently, the overall body of evidence should be interpreted cautiously.

This scoping review provides a comprehensive overview of the current evidence regarding the relationship between vitamin D status and neonatal sepsis. Most available studies are observational and geographically concentrated, with many reporting an association between vitamin D deficiency and an increased risk of neonatal sepsis, particularly among preterm infants and LOS cases. However, the substantial heterogeneity across studies, including differences in study design, patient populations, diagnostic criteria for sepsis, and laboratory methods, limits the comparability of findings and prevents definitive conclusions.

One of the main methodological challenges was inconsistencies in vitamin D assessment. Different analytical techniques, cut-off values for deficiency, and timing of blood sampling were used. Immunoassays were most commonly used, despite their susceptibility to inter-assay variability and measurement bias, whereas the more accurate LC-MS/MS reference method was used less frequently. These differences emphasize the need for standardized protocols in future research. An additional challenge is the lack of a universally accepted threshold for vitamin D deficiency. Across the included studies, deficiency was defined using cut-off values ranging from <10 ng/mL to <30 ng/mL. Such variability substantially limits direct comparison of study findings, contributes to inconsistent estimates of vitamin D deficiency prevalence, and may partly explain the conflicting associations reported between vitamin D status and neonatal sepsis.

Gestational age and birth weight represent particularly important confounding variables because preterm and extremely low birth weight infants are both more likely to have vitamin D deficiency and a substantially increased baseline risk of neonatal sepsis. Although several observational studies performed multivariable analyses, adjustment for gestational age, birth weight, maternal vitamin D status, and other neonatal risk factors was inconsistent across the literature. Consequently, residual confounding cannot be excluded and may partly account for the observed associations. Furthermore, the limited number of randomized controlled trials restricts the ability to establish a causal relationship between vitamin D deficiency and neonatal sepsis.

Vitamin D supplementation studies were limited and heterogeneous. Although some studies suggest possible protective effects, variations in supplementation regimens, timing of administration (maternal versus neonatal), and study methodology make the results difficult to interpret. Therefore, the current evidence is insufficient to support routine vitamin D supplementation, specifically for the prevention of neonatal sepsis. Future well-designed randomized controlled trials with standardized supplementation protocols and clearly defined outcomes are warranted.

The currently available evidence also does not allow conclusions regarding the optimal source or formulation of vitamin D for preventing neonatal sepsis. The included studies did not compare dietary vitamin D intake, different supplement formulations, or pharmacological preparations. Therefore, these aspects remain beyond the scope of the current review and require dedicated clinical investigation.

The biological plausibility of this association is supported by experimental studies demonstrating the role of vitamin D in regulating innate and adaptive immune responses, including antimicrobial peptide production, cytokine regulation, and inflammatory signaling pathways. Some studies have also reported associations between vitamin D status and inflammatory biomarkers, although these findings are inconsistent across the literature.

The strengths of this review include its comprehensive literature search, inclusion of a broad range of study designs, and systematic identification of methodological gaps. However, this review has several limitations. The included studies were restricted to English-language publications, and, consistent with the scoping review methodology, no formal assessment of methodological quality or risk of bias was performed. In addition, most of the included studies had an observational design, limiting the ability to infer causal relationships between vitamin D deficiency and neonatal sepsis. Considerable heterogeneity existed in the definitions of vitamin D deficiency, laboratory methods used to measure 25-hydroxyvitamin D, and diagnostic criteria for neonatal sepsis, reducing comparability across studies. Furthermore, relatively few studies evaluated long-term clinical or neurodevelopmental outcomes. Overall, this review highlights important gaps in the current evidence and underscores the need for methodological standardization and high-quality multicenter prospective studies to better understand the role of vitamin D in neonatal sepsis.

### 4.2. Future Perspectives

Future research should prioritize adequately powered multicenter prospective studies using standardized vitamin D assay methodologies, uniform definitions of vitamin D deficiency, and harmonized diagnostic criteria for neonatal sepsis. Studies should consistently adjust for relevant maternal and neonatal confounders, including gestational age, birth weight, maternal vitamin D status, nutritional factors, and illness severity. Well-designed randomized controlled trials are also required to determine whether maternal or neonatal vitamin D supplementation improves clinically meaningful outcomes, including sepsis incidence, mortality, duration of hospitalization, and long-term neurodevelopment, rather than merely confirming observational associations.

## 5. Conclusions

The current body of evidence suggests a possible association between lower vitamin D concentrations and neonatal sepsis; however, this evidence is derived predominantly from observational studies and therefore cannot establish causality. Reverse causation and residual confounding remain plausible explanations for at least part of the observed associations and should be considered when interpreting the available evidence. Vitamin D influences the innate and adaptive immune responses, including the regulation of antimicrobial peptides and inflammatory pathways, which may be particularly relevant in neonatal immune imbalances. Despite these findings, the available evidence remains insufficient to establish a causal relationship. Future randomized trials should be based on a clear and standardized sepsis definition that can better clarify the temporal relationships and account for key confounders. Additionally, mechanistic studies are needed to further clarify the immunological pathways through which vitamin D influences susceptibility to infection in neonates. Importantly, randomized controlled trials are essential to evaluate the efficacy and safety of vitamin D supplementation in both mothers and neonates, particularly in high-risk populations, such as preterm infants. Current evidence suggests that vitamin D deficiency is associated with neonatal sepsis; however, owing to the predominantly observational nature of the available studies, causality cannot be established. Residual confounding and reverse causation should also be considered when interpreting these findings. Supplementary Materials.

## Figures and Tables

**Table 1:** Study eligibility criteria for full-text screening.

	Inclusion Criteria	Exclusion Criteria
Populations of interest	Full-term neonates ≤ 28 days of life Preterm neonates ≤ 28 days of life	Studies involving populations other than neonates (e.g., adults, older infants, or animal models)
Interventions or exposures of interest	Blood levels of vitamin D e.g., serum 25-hydroxyvitamin D [25(OH)D] concentration Vitamin D supplementation (dose, duration, and regimen)	Studies that do not report circulating vitamin D levels or details on supplementation
Outcomes of interest	Risk/incidence of early-onset sepsis (EOS, occurring within ≤72 h of birth) and/or late-onset sepsis (LOS, occurring >72 h after birth)	Studies that do not investigate neonatal sepsis as a primary or secondary outcome
Study designs of interest	Empirical studies e.g., randomized controlled trials (RCTs), cohort studies, case-control studies, case series, case reports and cross-sectional studies (incl. study protocols via ClinicalTrials.gov) Systematic and scoping reviews and meta-analyses that provide relevant data Studies must pertain to humans; may be conducted in any country Only peer-reviewed evidence sources	Non-empirical studies e.g., expert opinions, commentary or debate papers, editorials, letter to the editor, or conference abstracts without full data Study of animals, cells, or other non-human subjects Unpublished studies e.g., conference abstracts
Language of interest	English	Other than English
Timeframe of publication	No time restrictions on publication date	

## Data Availability

No new data were created or analyzed in this study. Data Sharing is not applicable to this article.

## Supplementary Materials

The following supporting information can be downloaded at: https://www.mdpi.com/article/10.3390/nu18152517/s1, Supplementary File S1. PRISMA-ScR Checklist, manuscript ID 4419551; Supplementary File S2. Detailed description of the selected studies. Full table.

## Abbreviations

The following abbreviations are used in this manuscript:

ELBW	Extremely low birth weight
EOS	Early-onset sepsis
LOS	Late-onset sepsis
LMIC	Low- and middle-income countries
NICU	Neonatal intensive care units
PRISMA-ScR	Preferred Reporting Items for Systematic Reviews and Meta-Analyses Extension for Scoping Reviews
VDR	Vitamin D receptor

## Appendix A

### Search Strategy

**Table A1:** MEDLINE (via PubMed) 26 November 2025.

Concept, Domain	Search Term, Query
MeSH terms for: **vitamin D**	“Vitamin D”[Mesh] OR “Vitamin D Deficiency”[Mesh]
Natural terms for: **vitamin D** (title/abstract/keywords)	“vitamin D”[tiab] OR “hypovitaminosis D”[tiab] OR cholecalciferol[tiab] OR 25-hydroxyvitamin[tiab] OR 25-hydroxycholecalciferol[tiab]
MeSH terms for: **sepsis**	“Sepsis”[Mesh]
Natural terms for: **sepsis** (title/abstract/keywords)	sepsis[tiab]
MeSH terms for: **neonate**	“Infant”[Mesh]
Natural terms for: **neonate** (title/abstract/keywords)	Infant*[tiab] OR newborn*[tiab] OR neonate*[tiab] OR neonatal[tiab] OR postnatal[tiab]
MeSH terms for: **neonate + sepsis**	“Neonatal Sepsis”[Mesh]
Combined search blocks	(#1 OR #2) AND {[(#3 OR #4) AND (#5 OR #6)] OR #7}
Search filter	NOT (“animals”[Mesh] NOT “humans”[Mesh])

Total search, items found: **98**

(“Vitamin D”[Mesh] OR “Vitamin D Deficiency”[Mesh] OR “vitamin D”[tiab] OR “hypovitaminosis D”[tiab] OR cholecalciferol[tiab] OR 25-hydroxyvitamin[tiab] OR 25-hydroxycholecalciferol[tiab]) AND (((“Sepsis”[Mesh] OR sepsis[tiab]) AND (“Infant”[Mesh] OR Infant*[tiab] OR newborn*[tiab] OR neonate*[tiab] OR neonatal[tiab] OR postnatal[tiab])) OR “Neonatal Sepsis”[Mesh]) NOT (“animals”[Mesh] NOT “humans”[Mesh]).

**Table A2:** EMBASE 26 November 2025.

Concept, Domain	Search Term, Query
Emtree terms for: **vitamin D**	‘vitamin D’/exp OR ‘vitamin D deficiency’/exp
Natural terms for: **vitamin D** (title/abstract/keywords)	‘vitamin d’:ti,ab,kw OR ‘hypovitaminosis d’:ti,ab,kw OR ‘cholecalciferol’:ti,ab,kw OR ‘25-hydroxyvitamin’:ti,ab,kw OR ‘25-hydroxycholecalciferol’:ti,ab,kw
MeSH terms for: **sepsis**	‘sepsis’/exp
Natural terms for: **sepsis** (title/abstract/keywords)	‘sepsis’:ti,ab,kw
MeSH terms for: **neonate**	‘infant’/exp
Natural terms for: **neonate** (title/abstract/keywords)	‘infant*’:ti,ab,kw OR ‘newborn*’:ti,ab,kw OR ‘neonate*’:ti,ab,kw OR ‘neonatal’:ti,ab,kw OR ‘postnatal’:ti,ab,kw
MeSH terms for: **neonate + sepsis**	‘newborn sepsis’/exp
Combined search blocks	(#1 OR #2) AND {[(#3 OR #4) AND (#5 OR #6)] OR #7}
Search filter	NOT (‘conference abstract’/it OR ‘conference review’/it) NOT (‘animal’/exp NOT ‘human’/exp)

Total search, items found: **385**

(‘vitamin D’/exp OR ‘vitamin D deficiency’/exp OR ‘vitamin d’:ti,ab,kw OR ‘hypovitaminosis d’:ti,ab,kw OR ‘cholecalciferol’:ti,ab,kw OR ‘25-hydroxyvitamin’:ti,ab,kw OR ‘25-hydroxycholecalciferol’:ti,ab,kw) AND (((‘sepsis’/exp OR ‘sepsis’:ti,ab,kw) AND (‘infant’/exp OR ‘infant*’:ti,ab,kw OR ‘newborn*’:ti,ab,kw OR ‘neonate*’:ti,ab,kw OR ‘neonatal’:ti,ab,kw OR ‘postnatal’:ti,ab,kw)) OR ‘newborn sepsis’/exp) NOT (‘conference abstract’/it OR ‘conference review’/it) NOT (‘animal’/exp NOT ‘human’/exp).

## Appendix B

### Excluded Articles with Reasons for Exclusion: Described Full-Text Article Could Not Be Retrieved Despite Additional Searches in PubMed, EMBASE, Google Scholar, and Publisher Websites

**Table A3:** Excluded Articles and Reasons for Exclusion.

Author, Year	Title	Reasons for Exclusion
Delrue C et al., 2023	Vitamin D deficiency: an underestimated factor in sepsis?	Wrong patient population
Anand Asati A et al., 2023	To determine the correlation of 25-OH Vitamin D levels between newborns with their mothers in case of sepsis.	No article found
Prasad R et al., 2018	Vitamin D levels in late pre-term neonates and its association with sepsis	No article found
Yi X et al., 2023	Neonatal 25-hydroxy vitamin D levels after birth and 2 to 4 weeks after vitamin D supplementation and their impacts on complications.	Due to language restrictions
Eren E et al., 2025	Effect of 1,25-dihydroxy vitamin D3 on inflammation, antimicrobial peptide, and D-dimer levels in Escherichia coli-induced sepsis in neonatal calves.	Wrong patient population
Grant WB, 2010	Vitamin D supplementation of mother and infant could reduce risk of sepsis in premature infants.	Wrong study design
Yang LR et al., 2016	Relationship between vitamin D deficiency and early-onset neonatal sepsis.	Due to language restrictions
Shuang L et al., 2019	Effects of fat-soluble vitamins supplementation in early life on common complications and neural development in very low birth weight infants.	Wrong intervention
Agrawal P et al., 2023	To determine the maternal and neonatal plasma vitamin D levels and the possible effect of the severity of vitamin D deficiency on early onset sepsis.	No article found
Asati AA et al., 2023	To evaluate the impact of vitamin D status at birth and in the mother’s plasma on the risk of sepsis.	No article found
Grant WB, 2010	Vitamin D supplementation could reduce risk of sepsis in infants.	Wrong study design
Abdelmaksoud SR et al., 2021	Lower vitamin D level as a risk factor for late onset neonatal sepsis: an observational case-control study.	No article found
Tekgündüz KŞ et al., 2015	Is vitamin D deficiency alone sufficient to increase the incidence of neonatal sepsis?	Wrong study design
Elbehery MYM et al., 2025	Serum 25-Hydroxyvitamin D concentration status and neonatal immune function: new perspectives in anticipating late onset sepsis among preterm neonates at tertiary care centres (a prospective study).	No article found
