# Supplementary material for: Vitamin D and Neonatal Sepsis: Current Evidence, Biological Mechanisms, and Future Directions—A Scoping Review
Source: Nutrients. 2026 Aug 3;18(15):2517. doi: 10.3390/nu18152517 (PMC13468460; doi:10.3390/nu18152517)
Supplement: Supplementary file 1 [file nutrients-18-02517-s001.zip › Supplementary File S2. Detailed description of the selected studies. Full table..pdf]

Appendix C. Comprehensive characteristics of studies included in the scoping review.

Number [reference]	Author(s)	Year	Country of origin	Study design	Study objective	Adjustment for confounders	Sample size (if reported)	Gestational age criteria / definitions	Type of sepsis studied	Sepsis definition used in study	Vitamin D exposure	Biological sample used for vitamin D assessment	Timing of vitamin D assessment	Method of serum 25(OH)D measurement	Dose of vitamin D	Duration of supplementation	Route of administration	Serum 25(OH)D concentration (ng/mL) *[ng/mL] x2,5 = [nmol/L]*	Cut-off values for 25(OH)D serum concentration (ng/mL) *[ng/mL] x2,5 = [nmol/L]*	Relationship between vitamin D and sepsis	Reported occurrence/ incidence of sepsis	Reported biomarkers or immune response measures	Contribution to knowledge over time / methodological advances / trends	Study limitations as reported by authors	Identified gaps in the literature	Additional comments / observations / issues with data
1 [22]	Çekmez et al.	2014	Turkey	Case-control study	To evaluate the diagnostic value of serum 25-hydroxyvitamin D level, uPAR, IL-33 and ST2 in neonatal sepsis and compare them with conventional inflammatory markers (CRP, TNF-α, IL-6).	No	60 neonates (culture-positive neonatal sepsis: 40; control group: 20)	Term and near-term neonates; gestational age assessed by last menstrual period, obstetric ultrasonography.	Late-onset (>72 h)	Neonatal sepsis classified according to criteria defined by Gitto et al. [23]; only infants with high probable sepsis and positive blood culture were included in the analysis.	Serum 25(OH)D level	Neonatal venous blood	Postnatal- neonates	Immunoassay	Not applicable	Not applicable	Not applicable	Control group: mean 35±19;<br>Sepsis (day 1): mean 69±7.5;<br>Sepsis (48-72h): mean 49±6.4;	Sufficiency: 20	Increased risk: Higher levels observed during acute sepsis compared with controls.	Case-control design; incidence not reported.	Serum 25(OH)D; CRP; TNF-α; IL-6; uPAR; IL-33; sST2	One of the first neonatal studies assessing dynamic changes in serum 25(OH)D during sepsis and comparing its diagnostic performance with both traditional and novel inflammatory biomarkers.	Not reported	Further studies are needed to understand the role and predictive value of 25(OH)D, uPAR, IL-33 and ST2 in the diagnosis and follow-up of neonatal sepsis.	The analysis included culture-positive, LOS, resulting in a highly selected study population; serial biomarker measurements allowed assessment of temporal changes during treatment; no maternal vitamin D levels or supplementation data were reported.
2 [24]	Cizmeci et al.	2015	Turkey	Case-control study	To investigate whether neonates with EOS have lower cord-blood 25(OH)D levels compared with healthy controls and whether low vitamin D status is associated with increased risk of EOS.	Yes (gestational age; birth weight; gender)	83 neonates (EOS: 40; control group: 43)	Neonates admitted ≤3 days of age with clinical and laboratory criteria for suspected EOS.	Early-onset (≤72 h)	Clinical signs plus laboratory criteria including: Töllner score ≥10; IL-6 ≥60 pg/ mL; I/T >20%; CRP (≥8 mg/L); blood cultures not routinely available.	Serum 25(OH)D level	Cord blood	Postnatal- neonates (at birth)	LC-MS/MS	Maternal supplementation recorded only descriptively	Not applicable	Oral (assumed; not explicitly stated)	EOS group: median 12.6 (3.1-78.9)<br>Control group: median 21 (5-118)	Deficiency: ≤20<br>Insufficiency: 21-29<br>Normal: ≥30	Increased risk: Low cord-blood 25(OH)D (<30 ng/mL) associated with higher EOS risk; Adjusted OR 5.6 (95% CI 1.3-23.5).	Case-control design; 53 suspected EOS cases among 2571 live births, 40 confirmed EOS.	IL-6; CRP; ANC	First controlled study evaluating cord-blood vitamin D status in relation to EOS; reflects increasing interest in non-skeletal, immunomodulatory roles of vitamin D in neonatal outcomes.	Observational design (no causality); no maternal serum 25(OH)D measurements; lack of blood culture EOS confirmation	Role of maternal supplementation in preventing EOS; need for randomized controlled trials.	Absence of maternal serum 25(OH)D measurements restricts interpretation of maternal-fetal vitamin D transfer; use of cord-blood 25(OH)D as a proxy for prenatal vitamin D exposure is appropriate and consistent with prior literature. Prematurity independently associated with increased EONS risk (OR 14.9; 95% CI 2.3-94.4).
3 [25]	Cetinkaya et al.	2015	Turkey	Non-randomised experimental study	To evaluate the association between maternal and neonatal 25-hydroxyvitamin D levels and the risk of early EOS in term infants, and to assess the effect of severity of vitamin D deficiency on EOS development.	Yes (season of birth; maternal vitamin D supplementation during pregnancy; maternal clothing habits; socioeconomic and educational status; gestational age; birth weight; Apgar scores)	100 neonates (EOS susp. group: 50; control group: 50)	Term infants ≥37 weeks gestation	Early-onset (≤72 h)	EOS defined by clinical signs and laboratory criteria by Gitto et al. [23] within the first 72 hours of life, including CRP, CBC, and blood culture results, categorized as highly probable, probable, possible, or no sepsis.	Serum 25(OH)D level<br>Vitamin D supplementation	Neonatal venous blood; Maternal blood	Prenatal- mothers; Postnatal- neonates (≤72 hours of life)	LC-MS/MS	Prenatal multivitamin supplementation including 500 IU/day	Categorised as: no use/ irregular use (<3 months)/ regular use (>3 months)	Oral	Neonates: EOS group: mean 8.6±3.1; control group: mean 19.0±4.8<br>Mothers: EOS group: mean 22.2±6.8; control group: mean 36.2±1.8	Severe deficiency: <10<br>Insufficiency: 11-32<br>Adequacy: 32-100	Increased risk: Lower maternal and neonatal 25(OH)D levels associated with increased risk of EOS.	50 term infants with highly probable EOS identified during study period.	CRP; WBC; ANC	Highlights prenatal vitamin D status as a potentially modifiable risk factor for EOS.	Not reported	Lack of randomized controlled trials; limited data in preterm neonates; unclear optimal vitamin D supplementation dose for EOS prevention.	Results align with emerging evidence on vitamin D and neonatal immunity.
4 [26]	Say et al.	2017	Turkey	Case-control study	To evaluate whether low vitamin D levels in cord blood are associated with neonatal sepsis in preterm infants.	Yes (gestational age; birth weight; cord blood vitamin D level)	117 neonates; 100 infants included in final analysis	Gestational age <37 weeks	Early-onset (≤7 days of life); Late-onset (day 7-30 of life)	Clinical suspicion supported by laboratory abnormalities and confirmed when blood cultures were positive.	Not reported	Cord blood	Postnatal- neonates (at birth)	Immunoassay	Not applicable	Not applicable	Not applicable	Exact values not reported; distribution reported by categories: concentration of vitamin D ≤5: 63%; 5-15: 24%; ≥15: 13%	Severe deficiency: <5<br>Insufficiency: 5–15<br>Sufficiency: ≥15	No association: Low cord-blood 25(OH)D levels as <5 ng/mL was not associated with the occurrence of both EOS and LOS in the model (RR, 0.87; 95% CI, 0.73-1.03; p=0.11 and RR, 1.01; 95% CI, 0.86-1.19; p=0.84)	Case-control design; incidence not reported.	IL-6; CRP; WBC; ANC; I/T ratio	Reported as first study evaluating this association specifically in preterm infants; adds evidence questioning vitamin D as an independent sepsis risk factor.	Maternal vitamin D levels not measured; no dietary data influencing vitamin D status; seasonal limitation (summer births only); limited sample size	Need for larger prospective studies; need for randomized controlled trials; better evaluation of maternal vitamin D influence.	Important distinction: deficiency not independent risk after adjustment.
5 [27]	Gamal et al.	2017	Egypt	Case-control study	To measure neonatal and maternal serum 25-hydroxyvitamin D levels in neonates with EOS and to assess their relationship with sepsis markers.	Yes (gestational age, maternal risk factors, IL-6, CRP, birth weight, gender)	80 neonates (sepsis group: 50 neonates; control group: 30)	Preterm: mean gestational age: 34.1±1.26 weeks<br>Term: mean gestational age 37.5±0.98 weeks	Early-onset (≤72 h)	Clinical signs (≥2 systems affected) plus laboratory criteria (I/T ratio >0.2, toxic neutrophil granulation, WBC >25,000/mm³), occurring within <72 hours of life.	Serum 25(OH)D level	Neonatal venous blood; Maternal blood	Prenatal- mothers; Postnatal- neonates (at birth)	Immunoassay	Not applicable	Not applicable	Not applicable	Neonates: sepsis group 2.6±0.7;<br>control group: 9.8±0.9<br>Mothers: sepsis group: 17±8.3;<br>control group: 20.2±8.6	Deficiency: <12<br>Insufficiency: 12-20<br>Sufficiency: >20<br>EOS prediction cut-off: neonatal <8; maternal <16.8	Increased risk: Significant negative correlations between neonatal and maternal 25-OH vitamin D serum levels and all sepsis markers and significant positive correlations between neonatal and maternal 25-OH vitamin D levels were present.	Case-control design; incidence not reported.	CRP; IL-6; I/T ratio; ANC; WBC; PLT	Supports emerging evidence linking neonatal and maternal vitamin D deficiency with EOS and proposes 25(OH)D as a potential early predictive biomarker.	Small sample size; maternal vitamin D measured only at delivery; no assessment of vitamin D binding protein; no analysis of causative pathogens	Lack of longitudinal maternal vitamin D data; need for interventional supplementation trials; and pathogen-specific analyses in neonatal sepsis.	A major strength is the simultaneous assessment of maternal-neonatal vitamin D status, which aligns well with the PCC framework. Preterm neonates had significantly lower neonatal and maternal 25(OH)D levels and higher vulnerability to EOS.
6 [28]	Zheng et al.	2018	China	Randomised controlled trial	To investigate the effect of vitamin D on apoptosis of T-lymphocyte subsets and inflammatory factors in neonatal sepsis and explore possible immunological mechanisms.	No	250 neonates (sepsis: 150: (vitamin D treatment: 75; treatment control group: 75); healthy control group: 100)	Not reported	Both	Neonatal sepsis confirmed by positive blood culture and supportive imaging findings.	Serum 25(OH)D level<br>Vitamin D supplementation	Neonatal venous blood	Postnatal- neonates (before treatment and after 72h of treatment)	Immunoassay	0.25 µg	At least 72 hours (dose reduced after stabilization)	Intramuscular injection	Observation group: 22.52 ± 5.56 mg/L<br>Treatment control group: 14.85 ± 6.14 mg/L<br>Healthy control group: 26.38 ± 6.56 mg/L	Median used for analysis: 15.82 mg/L (no standard deficiency/sufficiency thresholds defined)	Protective: Higher vitamin D associated with lower mortality and improved immune markers.	Not reported (only enrolled cases)	CD4+ T-lymphocytes; CD8+ T-lymphocytes; TNF-α; IL-1; calcitonin	Provides experimental clinical evidence on immunomodulatory effects of vitamin D in neonatal sepsis; explores mechanistic immune markers rather than only epidemiological association.	Limited sample size; potential dosing concerns (risk of hypercalcemia); vitamin D not confirmed as independent risk factor	Lack of large, multicenter trials; need for optimal dosing, timing, and administration strategy; need for mechanistic in-vitro studies.	Study includes only culture-proven sepsis cases; gestational age and EOS vs LOS not stratified; strong focus on immunological endpoints rather than incidence.
7 [29]	El-Kader et al.	2018	Egypt	Case-control study	To investigate the association between vitamin D level and inflammatory cytokines (IL-6, CRP) in full-term neonates with early-onset sepsis.	No	50 neonates (EOS: 25; control group: 25)	Term neonates (≥37 weeks gestation)	Early-onset (≤72 h)	Combined clinical and laboratory diagnosis according to Gitto et al. [23], high probable EOS based on clinical and laboratory findings.	Serum 25(OH)D level	Neonatal venous blood; Maternal blood	Prenatal- mothers; Postnatal- neonates (≤72 hours of life)	Immunoassay	Not applicable	Not applicable	Not applicable	Neonates: EOS group: mean 8.56±2.18;<br>control group: 28.55±3.05; p<0.001<br>Mothers: EOS group mean: 22.30±5.05;<br>control group: 36.05±1.24; p<0.001	Severe deficiency: <10<br>Insufficiency: 11-32<br>Adequate: 32-100<br>Neonatal EOS cut-off: ≤11.5	Increased risk: Lower maternal and neonatal 25(OH)D levels associated with increased risk of EOS.	Case-control design; incidence not reported.	IL-6; CRP; serum 25(OH)D	Supports emerging evidence linking low neonatal vitamin D levels with increased risk of EOS and inflammatory activation.	Small sample size; single-center study	Need for larger multicenter studies and meta-analyses to confirm findings.	Vitamin D showed a strong inverse correlation with IL-6 but not with CRP; neonatal vitamin D level differed significantly between culture-proven and culture-negative EOS; findings limited to full-term neonates and EOS only.
8 [30]	Dhandai et al.	2018	India	Prospective observational study	To assess vitamin D deficiency as a possible risk factor for LOS in term and late preterm neonates and to examine the correlation between maternal and neonatal vitamin D levels during the neonatal period.	No	120 neonates (LOS: 60; control group: 60)	Term: 37-42 weeks gestation<br>Late preterm: 35-36+6 weeks gestation	Late-onset (>72 h)	LOS defined as development of signs and symptoms of severe sepsis after 72 hours of life with a positive sepsis screen (≥2 positive parameters according to Indian National Neonatology Forum guidelines).	Serum 25(OH)D level	Neonatal venous blood; Maternal blood	Prenatal- mothers; Postnatal- neonates (>72 hours of life)	Immunoassay	Not applicable	Not applicable	Not applicable	Neonates: LOS group: mean 15.4 (SD 10.0; range 1.75-50.0); control group: mean 21.4 (SD 9.5; range 3.7-35.1)<br>Mothers: LOS group: mean 17.9 (SD 11.9);<br>control group: mean 23.6 (SD 9.5)	Deficiency: <20<br>Insufficiency: 20-29<br>Optimum: 30-50	Protective: Lower neonatal and maternal serum 25(OH)D levels were significantly associated with late-onset neonatal sepsis. Vitamin D-deficient neonates had higher odds of LOS (OR 1.7), with a strong correlation between maternal and neonatal vitamin D levels. No causal inference or independent effect was established.	Sixty cases of LOS identified during the study period.	CRP	One of the early prospective studies specifically examining the association between vitamin D deficiency and LOS.	Small sample size; no multivariable regression analysis; no adjustment for known LOS confounders; no seasonal stratification of vitamin D levels; vitamin D levels measured only during acute illness	Lack of randomised controlled trials on vitamin D supplementation for LOS prevention; uncertainty regarding optimal dose and timing of maternal or neonatal supplementation.	Observational design limits causal inference; strong correlation between maternal and neonatal vitamin D levels (Pearson R = 0.896).
9 [31]	Saboute et al.	2019	Iran	Case-control study	To evaluate the relationship between maternal serum 25-hydroxyvitamin D levels and EOS, considering maternal and neonatal risk factors.	Yes (maternal age, gestational age, Apgar score, perinatal complications, serum vitamin D level)	64 neonates (EOS: 32; control group: 32)	Term neonates (≥37 weeks gestation)	Early-onset (≤72 h)	EOS diagnosed within the first 72 hours of life based on clinical signs, laboratory findings (elevated CRP, hematologic indices), and/or positive blood culture.	Serum 25(OH)D level<br>Vitamin D supplementation	Maternal blood	Prenatal - mothers	Immunoassay	Maternal supplementation	Majority >3 months during pregnancy	Oral (assumed; not explicitly stated)	Mean 25.72±12.37(range 2-51.8)	Deficiency/ insufficiency: <30<br>Sufficiency: ≥30	Protective: Maternal serum 25(OH)D levels were significantly lower in the sepsis group compared with controls (p=0.03). Maternal vitamin D supplementation during pregnancy was associated with a significantly reduced risk of EOS (p=0.003).	Case-control design; incidence not reported.	CRP; cytokines discussed conceptually but not directly measured	Contributes to growing evidence linking maternal vitamin D status with neonatal infectious outcomes; supports data suggesting protective role of prenatal vitamin D against EOS.	Small sample size; single-center design; lack of pathogen-specific culture analysis; neonatal vitamin D levels not measured; no assessment of LOS	Need for large, multicenter prospective studies; randomized controlled trials on vitamin D supplementation and neonatal sepsis outcomes; evaluation of neonatal and cord blood vitamin D status.	Small, case-control study including only term neonates, limiting generalizability and causal inference; neonatal vitamin D status not assessed; vitamin D supplementation details and pathogen-specific sepsis data insufficiently reported.
10 [32]	Ozdemir et al.	2019	Turkey	Case-control study	To evaluate maternal and neonatal serum 25-hydroxyvitamin D levels and assess their association with the development of neonatal sepsis.	Yes (gestational age, birth weight, gender, season of birth, maternal vitamin D intake, sunlight exposure)	107 neonates (sepsis group: 51; control group: 56)	Term neonates (≥37 weeks gestation)	Both	EOS defined by clinical signs and laboratory criteria by Gitto et al. [23] within the first 72 hours of life, including CRP, CBC, and blood culture results, categorized as highly probable, probable, possible, or no sepsis.	Serum 25(OH)D level	Neonatal venous blood; Maternal blood	Prenatal- maternal; Postnatal- neonates	Immunoassay	Maternal supplementation (1200 IU/day)	From the first trimester until six months after delivery	Oral	Neonates (overall): mean 12.4±8.5;<br>Sepsis group: mean 11.0±5.5;<br>Control group: mean 13.8±10.6;<br>Mothers (overall): mean 13.0±8.7	Deficiency: <12<br>Insufficiency: 12-20<br>Sufficiency: >20	Increased risk: Vitamin D deficiency is associated with an increased risk of neonatal infection.	Case-control design; incidence not reported.	CRP; WBC; PLT	Extends previous EOS-focused research by including both EOS and LOS and maternal vitamin D status in term neonates.	Exclusion of preterm infants; low socio-economic status of participants; smaller LOS subgroup compared to EOS	Lack of randomized controlled trials; unclear optimal vitamin D level for immune protection in neonates; limited data on LOS and vitamin D relationship.	Prospective single-center study; findings suggest association but do not establish causality; sepsis diagnosis combined clinical findings, hematologic scoring systems, and microbiological cultures; vitamin D supplementation was not an intervention but a background exposure, limiting causal inference.
11 [33]	Agrawal et al.	2019	India	Case-control study	To assess the association between vitamin D deficiency and LOS in term neonates.	Yes (gestational age, gender, weight, maternal age, sepsis, religion)	225 neonates (LOS: 175; control group: 50)	Term neonates (≥37 weeks gestation)	Late-onset (>72 h)	LOS defined as sepsis occurring after 72 hours of life, diagnosed based on clinical features and laboratory investigations (culture-proven).	Serum 25(OH)D level	Neonatal venous blood	Postnatal- neonates (>72 hours of life)	Not reported	Not applicable	Not applicable	Not applicable	LOS group: mean 12.28±6.11<br>Control group: mean 14.88±7.2	Severe deficiency: <5<br>Deficiency: 5-15<br>Insufficiency: 15-20<br>Sufficiency: >20	Increased risk: Vitamin D deficiency significantly more frequent in neonates with late-onset sepsis.	Case-control design; incidence not reported.	Not reported	Not reported	Vitamin D levels measured only postnatally in neonates; maternal vitamin D status was not assessed	Not reported	Large imbalance between cases and controls (175 vs 50); method of vitamin D assay not specified.
12 [34]	Youssef et al.	2019	Egypt	Cross sectional study	To investigate the association between neonatal 25-hydroxyvitamin D levels and the expression of lymphocyte activation markers (HLA-DR, CD69, CD25, CD45RA) on T-lymphocyte subpopulations, and to assess their impact on neonatal infection.	No	56 neonates	Gestational age calculated based on last menstrual period or first-trimester ultrasound.	Not specified	Alteration in at least 2 of the clinical signs: respiratory, cardiological, temperature, haemodynamic, gastrointestinal, neurological and metabolic plus elevated I/T >0.2, the presence of toxic neutrophil granules or a WBC >25×10^9/L at the time of evaluation.	Serum 25(OH)D level	Cord blood; Maternal blood	Prenatal- maternal; Postnatal- neonates (at birth)	Immunoassay	Not applicable	Not applicable	Not applicable	Not provide numerical mean, median, or range values of serum 25(OH)D concentrations for neonates or mothers.	Severe deficiency: <5<br>Moderate deficiency: 5-10<br>Mild deficiency: >10-20<br>Sufficiency: >20	Increased risk: Vitamin D deficiency is associated with a reduction of lymphocyte subsets and altered T-lymphocyte activation, which may contribute to an increased risk of neonatal infection.	7 of 43 neonates (16.27%) with 25-OHD deficiency developed sepsis; no cases of sepsis were reported in neonates with normal 25-OHD levels.	CD25; CD69; HLA-DR; CD45RA expression on CD4+ and CD8+ T-lymphocyte subpopulations	The study contributes evidence linking perinatal vitamin D status with neonatal immune competence and infection risk, emphasizing the maternal-neonatal vitamin D axis.	Small sample size; lack of data on maternal vitamin D supplementation during pregnancy	Need for larger-scale and interventional studies assessing whether maternal vitamin D supplementation can reduce neonatal infection and sepsis risk.	Observational design; causality cannot be inferred; neonatal sepsis assessed during post-discharge follow-up according to hospital protocol.
13 [35]	Zhang et al.	2019	China	Observational study (retrospective cohort)	To investigate whether vitamin D deficiency in the first trimester of pregnancy is associated with placental inflammation in high-risk singleton pregnancies.	Yes (maternal age; gestational age; weight; gender; season of blood sample collection; gestational diabetes mellitus; pregnancy-induced hypertension; intrahepatic cholestasis of pregnancy; PROM)	23,396 mother-infant pairs (2,648 high-risk pregnancies with placental pathology)	Preterm birth defined as <37 weeks gestation; Subcategories: <31 weeks; 31-34 weeks; 34-36+6 weeks; ≥37 weeks	Not specified (neonatal sepsis reported as an outcome)	Not clearly defined	Serum 25(OH)D level	Maternal blood	Prenatal- mothers (first trimester: 9-13 weeks gestation)	Immunoassay	Not applicable	Not applicable	Not applicable	Median (IQR): placental inflammation negative group: 16.68 (12.1-21.2); placental inflammation positive group: 16.4 (11.8-20.5)	Severe deficiency: <8<br>Deficiency: <11<br>Insufficiency: 11-20<br>Sufficiency: ≥20	Increased risk: Severe vitamin D deficiency in early pregnancy was associated with placental inflammation. Maternal vitamin D levels were significantly lower in the inflammation group than those in the non-inflammation group (p=0.025).	placental inflammation negative: 0.5% placental inflammation positive: 2.4%	Placental histopathological inflammatory lesions	Provides large-scale human evidence linking severe early pregnancy vitamin D deficiency with placental inflammation; extends previous animal and mechanistic studies to clinical population data.	Retrospective study design; vitamin D levels only measured in first trimester; lack of longitudinal vitamin D assessment during pregnancy	Need for prospective longitudinal studies; need for mechanistic human studies linking vitamin D, placental inflammation, and neonatal immunity.	Neonatal sepsis analyzed as secondary outcome associated with placental inflammation, not as primary endpoint; vitamin D supplementation effects were not assessed; sepsis definition not explicitly standardized in manuscript.
14 [36]	Singh et al.	2020	India	Case-control study	To determine whether vitamin D status is affected in term neonates with EOS and its association with disease severity and outcome.	No	140 neonates (sepsis group: 70; control group: 70)	Term neonates (≥37 weeks gestation)	Early-onset (≤72 h)	Sepsis categorized as possible, probable, or highly probable based on number of clinical signs, CRP (>1mg/dL), hematological parameters, and blood culture results.	Serum 25(OH)D level	Neonatal venous blood	Postnatal- neonates (≤72 hours of life)	Immunoassay	Not applicable	Not applicable	Not applicable	Sepsis group: mean 16.00 (SD 10.49);<br>Control group: mean 29.07 (SD 8.36)	Severe deficiency: <11<br>Insufficiency: 11-32<br>Adequate: >32-100	Increased risk: Neonatal vitamin D level was lower in all seasons (winter, summer and monsoon) in the study group (p=0.02, 0.03 and 0.04, respectively). Higher mortality associated with severe vitamin D deficiency (p=0.005).	Case-control design; incidence not reported.	CRP; WBC; ANC; PLT	Adds evidence supporting an association between neonatal vitamin D deficiency and EOS severity and mortality in term neonates; supports growing observational evidence linking vitamin D status with neonatal sepsis outcomes.	Maternal vitamin D status was not evaluated	Need for studies assessing maternal-neonatal vitamin D relationship.	Study limited to term neonates and EOS; seasonal variation in vitamin D levels was observed but not adjusted for analytically.
15 [37]	Hagag et al.	2020	Egypt	Case-control study	To evaluate the therapeutic value of vitamin D supplementation as an adjuvant therapy in neonates with sepsis.	No	90 neonates (sepsis: 60 (group I (antibiotics only): 30; group II (antibiotics + vitamin D): 30); control group: 30)	Mean gestational age ≈ 36–37 weeks<br>No explicit gestational age cut-off provided	Early-onset (≤72 h)	Clinical syndrome in infant aged ≤28 days, manifested by systemic signs of infection with the isolation of bacterial pathogen from blood stream; clinical sepsis score based on: clinical signs; CRP; hematological parameters (WBC, ANC, PLT, I/T).	Serum 25(OH)D level<br>Vitamin D supplementation	Neonatal venous blood	Postnatal- neonates	Immunoassay	800 IU/day of vitamin D3	2 weeks	Oral	Group I: mean 16.95±2.60<br>Group II: mean 17.15±2.88<br>Controls: mean 24.36±3.35	Deficiency: ≤20	Protective: Vitamin D supplementation improved sepsis score and reduced inflammatory markers.	Case-control design; all cases were clinically diagnosed neonatal sepsis (suspected and confirmed).	hs-CRP; I/T ratio	One of the first randomized studies evaluating vitamin D as adjuvant therapy in neonatal sepsis; demonstrates therapeutic (not only associative) role of vitamin D.	Small sample size; heterogeneous population (suspected and confirmed sepsis)	Need for larger randomized controlled trials; lack of standardized vitamin D dosing protocols in neonatal sepsis.	Study focuses primarily on EOS; no adjustment for maternal vitamin D status or seasonality.
16 [38]	Bilgin et al.	2020	Turkey	Case-control study	To determine the relationship between maternal and neonatal serum 25-hydroxyvitamin D levels and community-acquired LOS and to determine a serum 25(OH)D cut-off predicting LOS.	No	92 neonates (LOS: 46; control group: 46)	Term neonates (≥37 weeks gestation)	Late-onset (>72 h)	Combined clinical and laboratory criteria according to European Medicines Agency sepsis score: clinical sepsis based on ≥2 clinical criteria and ≥2 laboratory criteria; blood, urine, and cerebrospinal fluid cultures obtained before antibiotic treatment (culture-positive and culture-negative sepsis).	Serum 25(OH)D level	Neonatal venous blood; Maternal blood	Postnatal- neonates (>72 hours of life; within 72 hours of late-onset sepsis diagnosis)	Immunoassay	Maternal supplementation reported as none/ irregular/ regular	Not reported	Oral	Neonates: sepsis group: 12.9±6.36;<br>control group: 21.0±6.38<br>Mothers: sepsis group: 9.8±6.07;<br>control group: 15.3±4.7	Deficiency: ≤ 20	Increased risk: Serum 25(OH)D levels of infants and their mothers in the study group were significantly lower than the control group (p<0.001). There was a positive correlation between 25(OH)D levels of mothers and infants in both groups (p<0.001).	Case-control design; incidence not reported.	CRP; WBC; I/T ratio; cathelicidin	Provides evidence supporting an association between low vitamin D levels and LOS in term infants, extending findings previously reported for EOS.	Vitamin D levels measured only during infection, not before or after; control group consisted of infants with hyperbilirubinemia; low rate of culture-proven sepsis; small sample size	Need for larger prospective and randomized studies evaluating vitamin D supplementation and neonatal infection outcomes.	Study limited to term neonates and community-acquired LOS; findings may not be generalizable to preterm infants.
17 [39]	Matejek et al.	2020	Czech Republic	Cohort study	Compare clinical outcomes of VLBW preterm infants with severe vitamin D deficiency (<10 ng/dL) vs >10ng/dL in umbilical cord blood. Evaluate umbilical cord vitamin D as a risk factor for respiratory distress syndrome.	Yes (gestational age)	81 VLBW neonates (<1500g)	Median gestation age 29 weeks (IQR 27–30)	Both	Sepsis was classified as either culture-proven or clinically suspected and treated with antibiotics for at least 5 days.	Serum 25(OH)D level	Cord blood; Neonatal venous blood; Maternal blood	Prenatal- maternal; Postnatal- neonates	LC-MS/MS	800-1000 IU/day (vitamin D2 parenteral, then vitamin D3 enteral)	Not reported	Enteral; Parenteral	Umbilical cord blood: median (IQR) 11.6 (6.8-19.2)<br>Maternal: median (IQR) 15.2 (9.6-26.4)<br>Neonatal at discharge: median (IQR) 19.2 (14.8-25.2)	Severe deficiency: <10<br>Deficiency: <20<br>Sufficiency: >20	No association: No statistically significant difference was observed.	Not reported.	Not reported	Contributes prospective cohort data to limited evidence base on vitamin D and non-skeletal outcomes in preterm infants; uses LC–MS/MS with exclusion of C3 epimers (methodological strength).	Non reported	Lack of consensus on optimal vitamin D levels in preterm neonates; need for larger, multicenter studies and randomized controlled trials.	High prevalence of vitamin D deficiency despite supplementation; sepsis outcomes reported but not primary focus of study; suggest no association between severe vitamin D deficiency and neonatal sepsis.
18 [40]	Behera et al.	2020	India	Cohort study	To evaluate the effect of maternal and neonatal vitamin D levels on the risk of culture-positive neonatal sepsis.	Yes (gestational age, postnatal age, gender)	80 neonates (sepsis: 40; control group: 40)	Term neonates (≥37 weeks gestation)	Both	Neonatal sepsis confirmed by positive blood culture in neonates admitted with clinical suspicion of sepsis.	Serum 25(OH)D level	Neonatal venous blood; Maternal blood	Prenatal- maternal; Postnatal- neonates	Immunoassay	Not applicable	Not applicable	Not applicable	Neonates: sepsis group: 12.71±2.82; control group): 25.46±7.02<br>Mothers: sepsis group: 20.92±3.92; control group: 27.31±6.83	Deficiency: <12<br>Insufficiency: 12-20<br>Sufficiency: >20	Increased risk: Neonates with vitamin D deficiency/insufficiency are at higher risk for developing sepsis than those with sufficient vitamin D levels. Lower vitamin D levels in mothers is also associated with increased risk of sepsis in the neonates.	Study included only neonates diagnosed with sepsis; incidence not reported	Serum 25(OH)D; CRP	Provides prospective evidence from an Indian setting supporting an association between low maternal/neonatal vitamin D levels and increased risk of culture-positive neonatal sepsis.	they did not study whether there is any variation in vitamin D level between culture-positive sepsis and clinical sepsis	Further large trials evaluating the effects of vitamin D supplementation on neonatal sepsis are required.	Vitamin D status was assessed using a single measurement at admission, with no longitudinal follow-up of vitamin D levels.
19 [41]	Choudhury et al.	2020	India	Prospective observational study	To determine the prevalence of vitamin D deficiency in very low birth weight preterm neonates at baseline and at 38±2 weeks post-menstrual age after daily supplementation with 800 IU vitamin D; to identify determinants of deficiency, assess growth outcomes, and evaluate vitamin D toxicity.	Yes (gestational age, weight, sex, maternal sun exposure, maternal vitamin D supplementation, morbidities, ventilation, sepsis, caffeine use)	83 neonates (101 enrolled; 83 completed follow-up)	<32 weeks gestational age; very low birth weight (VLBW)	Late-onset (>72 h)	Late-onset neonatal sepsis diagnosed according to unit clinical protocols (specific diagnostic criteria not explicitly defined).	Serum 25(OH)D level<br>Vitamin D supplementation	Neonatal venous blood	Postnatal- neonates	Immunoassay	800 IU/day	From enrollment until 38±2 weeks PMA	Oral	Reported as categories (deficiency, insufficiency, sufficiency); exact mean/median values not consistently provided	Deficiency: <20<br>Insufficiency: 21-29<br>Sufficiency: >30<br>Severe deficiency: <10<br>Toxicity: ≥150	Increased risk: Sepsis associated with lower vitamin D levels at follow-up.	Sepsis reported as a neonatal morbidity; 42 of 83 neonates experienced sepsis.	Not reported	Confirms very high prevalence of vitamin D deficiency in VLBW neonates in low- and middle-income settings; supports effectiveness and safety of higher-dose supplementation; adds observational evidence linking neonatal morbidities (including sepsis) with poorer vitamin D status at follow-up.	Single-center study; no long-term follow-up beyond term-equivalent age; not powered to assess determinants of vitamin D deficiency	Limited mechanistic studies linking vitamin D and neonatal immunity; need for adequately powered trials assessing sepsis outcomes.	Study focuses primarily on vitamin D status and growth; sepsis analyzed as an associated morbidity rather than primary outcome; no standardized sepsis definition reported.
20 [42]	Workneh Bitew et al.	2021	Ethiopia	Systematic review and meta-analysis	To determine the effects of maternal and newborn cord blood vitamin D levels on neonatal sepsis and to compute the pooled prevalence of cord blood vitamin D deficiency among neonates.	No	Total of 18 studies included. Maternal vitamin D analysis: 675 participants. Cord blood vitamin D analysis: 748 neonates with sepsis and 573 controls.	Term neonates (≥37 weeks gestation); Preterm neonates (<37 weeks gestation)	Both	Neonatal sepsis within first 28 days of life including bloodstream infections, meningitis, and pneumonia.	Serum 25(OH)D level	Cord blood; Maternal blood	Prenatal- maternal; Postnatal- neonates	Not consistently reported in included studies	Not applicable	Not applicable	Not applicable	Maternal vitamin D mean difference: -8.57 (95% CI: -13.09, -4.05); cord blood mean difference: -8.78 (95% CI: -11.58, -5.99).	Deficiency: <20	Protective: Neonates born from mothers with low vitamin D levels had greater risk of sepsis (MD -8.57; 95% CI: -13.09, -4.05). Low cord blood vitamin D levels were also associated with neonatal sepsis (MD -8.78; 95% CI: -11.58, -5.99).	Case-control design; incidence not reported.	Cytokines	The study synthesized observational evidence from 2014-2019 and highlighted growing evidence linking neonatal sepsis with vitamin D deficiency.	High heterogeneity between studies; differences in units of measurement; incomplete findings; inconsistent reporting of vitamin D measures; some studies excluded due to missing mean/SD values	Need for randomized controlled trials to determine optimal timing, dosage, and mode of vitamin D supplementation; limited geographic representation of studies.	Data extraction reflects pooled findings from included studies rather than individual participant data; several included studies focused specifically on EOS in term neonates. EOS in term neonates was significantly associated with lower vitamin D levels.
21 [43]	Helmy et al.	2021	Egypt	Case-control study	To evaluate the diagnostic value of the early-onset sepsis risk calculator and vitamin D levels as markers for early diagnosis of early-onset neonatal sepsis among newborns >34 weeks gestation exposed to PROM.	No	100 neonates (septic group: 50; control group: 50)	Neonates >34 weeks gestation	Early-onset (≤72 h)	EOS diagnosed based on clinical findings, laboratory investigations (CBC, CRP), EOS calculator results, and blood culture (gold standard).	Serum 25(OH)D level	Neonatal venous blood	Postnatal- neonates (≤72 hours of life)	Immunoassay	Not applicable	Not applicable	Not applicable	Sepsis group: mean±SD: 8.57±9.14;<br>median (IQR): 4.0 (3.2-9.2)<br>Control group: mean±SD: 50.61±12.55;<br>median (IQR): 52.0 (38.5-57.0)	ROC-derived EOS prediction: 27 (sensitivity: 2%; specificity: 98%)	Increased risk: Lower vitamin D associated with higher sepsis risk.	Case-control design; incidence not reported.	CRP; WBC; I/T ratio	Study contributes to emerging evidence on vitamin D as an adjunct biomarker for neonatal sepsis risk stratification, particularly when combined with clinical risk calculators.	Equivocal EOS risk calculator results may delay clinical management; EOS risk calculator is not widely implemented and has inconsistencies in certain parameters, which may reduce sensitivity for culture-positive sepsis	Limited evidence on vitamin D as an adjunct biomarker.	Study focuses exclusively on neonates exposed to PROM >18 hours; vitamin D assessed only postnatally; strong diagnostic accuracy reported, but observational design limits causal inference. Lower gestational age and birth weight observed in septic group compared to controls.
22 [44]	Jeengar et al.	2021	India	Case-control study	To evaluate the association between neonatal serum vitamin D levels and EOS.	No	122 neonates (culture-proven EOS: 62; control group: 60)	Neonates 30-40 weeks gestation	Early-onset (≤72 h)	Sepsis screen was considered to be positive if two or more of following parameters were present- TLC <5,000/mm3, absolute neutrophil count less than reference range, and positive CRP. Cases were labelled as newborn with culture-proven EOS, admitted as a case of sepsis within 72h of birth.	Serum 25(OH)D level	Neonatal venous blood	Postnatal- neonates (≤72 hours of life)	Immunoassay	Not applicable	Not applicable	Not applicable	Cases: 18.5 ±7.41;<br>Controls: 29.2±8.46	Deficiency: <20<br>Insufficiency: 20-29<br>Sufficiency: ≥30<br>ROC-derived EOS prediction: 25	Increased risk: Risk of EOS increased 8 times in neonates with 25OH-D level <30ng/mL [OR]=8.2; 95% CI: 3.08-21.82; p=.000).	Case-control design; incidence not reported.	TLC; CRP	Including both preterm and term neonates; supporting growing evidence linking neonatal vitamin D deficiency and EOS risk; shows trend toward considering vitamin D as modifiable risk factor in neonatal sepsis.	Maternal vitamin D levels not measured; no assessment of seasonal effects or maternal supplementation; single-center study; limited generalizability	Need for multicenter studies; need to evaluate maternal-neonatal vitamin D relationship; need for supplementation trials.	Strong diagnostic definition due to culture-proven sepsis; lack of multivariate analysis limits causal inference; supports vitamin D screening hypothesis but intervention data lacking.
23 [45]	Dogan et al.	2021	Turkey	Case-control study	To evaluate maternal and neonatal vitamin D status and to investigate the association between neonatal 25-hydroxyvitamin D levels and the development of LOS in preterm infants.	Yes (gestational age; birth weight; duration of mechanical ventilation)	108 neonates (LOS: 42; control group: 66)	Preterm neonates (<37 weeks gestation)	Late-onset (>72 h)	Presence of clinical signs of sepsis with a positive blood culture occurring between 4 and 30 days after birth.	Serum 25(OH)D level	Neonatal venous blood; Maternal blood	Prenatal- maternal (within 4 hours after delivery); Postnatal- neonates (within 6 hours of life)	LC-MS/MS	Not applicable	Not applicable	Not applicable	Neonates: LOS group: median 8.1 (IQR 5-9.5)<br>control group: median 12.2 (IQR 8.5-15.4)<br>Mothers: LOS group: median 9.5 (IQR 8-13)<br>control group: median 14.1 (IQR 10.3-18)	Severe deficiency: <5<br>Insufficiency: 5-15<br>Normal (sufficiency): >15	Increased risk: Comparison of the rate of low 25(OH)D levels between groups revealed a significant difference (p=0.003). In the study group, the frequency of low 25(OH)D levels were higher, whereas in the control group, the frequency of normal 25(OH)D levels was higher.	Case-control design; incidence not reported.	Serum 25(OH)D; cathelicidin; defensins	One of the limited studies focusing exclusively on preterm infants; demonstrates a strong independent association between low neonatal vitamin D levels and LOS after adjustment for key confounders; provides ROC-derived threshold (≤9.5ng/mL) for LOS risk prediction.	Inclusion limited to culture-proven LOS only; small sample size; limited power for subgroup analyses; no analysis of pathogen-specific vitamin D associations	Need for randomized controlled trials; limited data on optimal vitamin D supplementation strategies for LOS prevention in preterm infants.	Strong methodological adjustment for major LOS confounders; vitamin D measured with LC-MS/MS, increasing measurement reliability.
24 [46]	Kamsiah et al.	2021	Indonesia	Cross sectional study	To assess the relationship between maternal vitamin D levels and clinical outcomes of neonatal sepsis (mortality, respiratory support, length of hospital stay, blood culture).	No	41 neonates	Preterm defined as <36 weeks gestation; term ≥36 weeks (as reported in results)	Both	Neonatal sepsis defined as invasive bloodstream infection occurring within the first month of life, with or without bacteremia.	Serum 25(OH)D level	Neonatal venous blood	Postnatal- neonates	Immunoassay	Not applicable	Not applicable	Not applicable	Not reported	Not reported	No association: Statistical test showed p=0.712 (95% CI=0.623-1.353). Therefore, the relationship between vitamin D levels and blood culture was insignificant.	Study included only neonates diagnosed with sepsis; incidence not reported.	Cathelicidin	Adds data from Southeast Asia on association between neonatal vitamin D deficiency and sepsis outcomes, highlighting respiratory support requirement as a significant outcome.	Not reported	Need for prospective studies and randomized trials assessing causality, standardized vitamin D cut-offs, and maternal-neonatal vitamin D interactions.	Vitamin D categorized dichotomously (deficient vs normal) without reported numerical thresholds; sepsis included both proven and probable cases.
25 [47]	Mahmoud et al.	2021	Egypt	Cohort study	To evaluate the role of vitamin D in LOS in term neonates and examine correlations between serum 25(OH)D levels, inflammatory markers, disease severity, nSOFA score, and mortality risk.	Not specified	228 neonates (suspected LOS: 52; confirmed LOS: 74; septic shock: 24; control group: 80)	Term neonates (≥37 weeks gestation)	Late-onset (>72 h)	LOS is defined as an event that met criteria: sepsis occurred after the third day of life, a single bacterial/fungal pathogen isolated from blood, cerebrospinal, pleural, peritoneal fluid or sputum, WBC <1000 or >50,000/µL, ANC >17,670/microL, I/T ≥0.2, and PLT <50,000/microL. Sequential assessment of CRP values and elevated serum levels of PCT.	Serum 25(OH)D level	Neonatal venous blood	Postnatal- neonates (>72 hours of life)	Immunoassay	Maternal supplementation recorded only descriptively	Not applicable	Not applicable	Control group: mean 29.78±9.55;<br>Suspected sepsis: mean 19.07±8.44;<br>Confirmed sepsis: mean 14.13±3.59;<br>Septic shock: mean 11.08±4.96	Normal: >20<br>Relative deficiency: 15–20<br>Absolute deficiency: <15<br>Study defined deficiency:<20	Increased risk: Lower vitamin D associated with greater disease severity and mortality. Patients with confirmed or suspected infection had lower 25(OH)D levels however, 25(OH)D levels were markedly lower in the patients with septic shock than other confirmed or suspected infectious (p<0.0001).	Occurrence of LOS among admitted neonates, including:148 term neonates diagnosed with LOS and 80 healthy neonates serving as controls.	CRP; PCT; WBC; ANC; PLT; I/T ratio	Supports growing evidence linking vitamin D deficiency to worse neonatal sepsis outcomes; introduces combined evaluation using vitamin D levels and organ dysfunction scoring.	No serial vitamin D measurements; maternal vitamin D levels not measured; nSOFA not globally validated	Need for larger multicenter studies; need to clarify mechanisms of vitamin D in neonatal sepsis; need to validate nSOFA and vitamin D as prognostic tools.	Well-defined LOS cohort with severity stratification; strong correlation between vitamin D deficiency and disease severity; observational design limits causal inference; vitamin D supplementation effects remain untested.
26 [48]	Moore et al.	2021	United States of America	Case report	To describe a case of a full-term newborn with late-onset hypocalcemic seizures and vitamin D deficiency in the setting of Klebsiella pneumoniae bacteremia and to highlight the importance of evaluating for sepsis in neonates presenting with hypocalcemic seizures.	Not applicable	1 neonate	Term neonates (exact gestational age not specified)	Late-onset (>72 h)	Blood culture-proven sepsis diagnosed after 72 hours of life.	Serum 25(OH)D level	Neonatal venous blood; Maternal blood	Prenatal- maternal; Postnatal- neonates	Not reported	2000 IU/day, reduced to 400 IU/day	Initiated on day of life 19; continued with 400 IU/day by day of life 85	Oral	Neonates: median 7.4<br>Mothers: median 9.4	Deficiency: <30	Inconclusive: Authors suggest vitamin D deficiency may increase susceptibility to infection but state further studies are needed.	A case of Klebsiella pneumoniae bacteremia.	Serum 25(OH)D	First reported neonatal case linking vitamin D deficiency, late-onset hypocalcemia, and Gram-negative bacteremia outside of critical illness; extends adult findings on hypocalcemia and sepsis to neonatal population	Single case report; cannot establish causality between vitamin D deficiency and sepsis	Lack of studies evaluating the role of vitamin D supplementation in infection prevention; limited understanding of mechanisms linking vitamin D deficiency, hypocalcemia, and neonatal sepsis.	Case report design limits generalizability; vitamin D measurement methodology not reported.
27 [49]	Ahmad et al.	2021	Pakistan	Non-randomised experimental study	To determine whether granulocyte-to-lymphocyte ratio differs among neonates with sepsis according to vitamin D status (deficiency, normal levels, hypervitaminosis D), and whether differs by outcome (survived vs expired).	No	109 neonates	Not reported	Not specified	Clinical manifestations of neonatal sepsis; blood culture performed, with culture-proven sepsis identified in 22% of cases.	Serum 25(OH)D level	Not reported	Not reported	Not reported	Not applicable	Not applicable	Not applicable	Not reported as mean/median values; only categorical distribution provided	Deficiency: <20<br>Normal: 20-50<br>Hypervitaminosis: >50	No association: No significant difference in granulocyte-to-lymphocyte ratio across vitamin D categories	Study included only neonates diagnosed with sepsis; 22% culture-proven sepsis.	GLR; IL-2; IL-6; IL-8; TNF-α	Adds evidence from a low-middle-income country setting suggesting no association between vitamin D status and inflammatory response among neonates with sepsis; contrasts with studies in other populations showing inverse associations.	Not reported	Not reported	Study focuses on inflammatory marker rather than direct sepsis risk or incidence; relevant mainly for mechanistic/inflammatory response context rather than preventive supplementation evidence.
28 [50]	Naseh et al.	2022	Iran	Case-control study	To evaluate the possible association between serum vitamin D levels in pregnant mothers and their neonates and the incidence of EOS, and to assess the association between maternal and neonatal serum vitamin D levels.	No	100 neonates (EOS: 50; control group: 50); 90 mothers (several delivered twins)	Neonates ≥34 weeks gestation	Early-onset (≤7 days of life)	Diagnosis of EOS based on clinical signs plus laboratory evaluation (including blood culture). Blood culture positive in 5 cases [10%]; negative in 45 cases [90%].	Serum 25(OH)D level	Neonatal venous blood; Maternal blood	Prenatal- maternal (last trimester of pregnancy); Postnatal- neonates	Not reported	Not applicable	Not applicable	Not applicable	Neonates: sepsis group: 9.97±4.09; range 3.3-25.2;<br>control group 9.04±2.91; range 2.2-14.4<br>Mothers: sepsis group: 22.0±8.84; control group: 27.3±8.49	Deficiency: <20<br>Ideal level: >30	Protective: Serum vitamin D levels were lower in mothers of the neonates with sepsis compared to the control mothers (55.01 ± 22.11 versus 68.24 ± 21.22 nmol/L, respectively; p=0.005). Compared to the sepsis cases, the control group had higher serum vitamin D levels (p=0.022).	Case-control design; incidence not reported.	CRP; WBC	Supports association between lower maternal and neonatal vitamin D levels and EOS; confirms positive correlation between maternal and neonatal vitamin D levels in healthy controls.	Limited sample size; matching based on gestational age range rather than exact GA; No stratification by confounders; unable to match for maternal vitamin D supplementation; maternal vitamin D measured during last trimester (not necessarily at delivery).	Need for larger studies; need to evaluate impact of vitamin D supplementation during pregnancy on EOS.	Majority of EOS cases were culture-negative (90%); the method used for serum 25(OH)D measurement was not reported; incidence rate of EOS was not calculated (case-control design).
29 [51]	Kumar et al.	2022	Pakistan	Cross sectional study	To evaluate the relationship between vitamin D deficiency and EOS and examine outcomes including respiratory support, length of hospital stay, and mortality.	No	41 neonates (60.9% with gestational age less than 36; 39.1% after 36 weeks of gestation)	Preterm defined as <36 weeks gestation; term ≥36 weeks	Early-onset (≤72 h)	Neonatal sepsis defined as invasive bloodstream infection occurring within the first month of life, with or without bacteremia.	Serum 25(OH)D level	Neonatal venous blood	Postnatal- neonates (≤72 hours of life)	Not reported	Not applicable	Not applicable	Not applicable	Not reported	Not reported	Inconclusive: No significant difference between the two groups (deficiency and normal vitamin D levels) in terms of mortality outcomes, blood culture, and length of hospital stay. However, we observed a positive correlation between vitamin D levels and the need for respiratory support.	Study included only neonates diagnosed with sepsis; incidence not reported.	CRP; cathelicidin	Not reported	Not reported	Need for prospective and interventional studies.	Study focuses exclusively on EOS; vitamin D deficiency associated with respiratory support requirement but not mortality or length of stay.
30 [52]	Ahmed et al.	2022	Pakistan	Cross sectional study	To ascertain the prevalence of vitamin D deficiency among cases of neonatal sepsis and to determine whether there is any association between vitamin D levels and mortality rate among neonates with sepsis.	No	110 neonates	Not reported	Not specified	Neonates admitted with clinical manifestations of neonatal sepsis; categorized as culture-proven sepsis (growth in blood, urine, or cerebrospinal cultures) or presumed sepsis (clinical signs without culture growth).	Serum 25(OH)D level<br>Vitamin D supplementation	Neonatal venous blood	Postnatal- neonates	Immunoassay	100,000 IU/ day intramuscular for 3 days, followed by 800 IU/ day	3 months	Intramuscular; oral	Overall median: 14.80	Deficiency: <20<br>Normal: 20-50<br>Hypervitaminosis: >50	No association	Study included only neonates diagnosed with sepsis; incidence not reported.	Serum 25(OH)D	Provides prevalence data on vitamin D deficiency among neonates with sepsis in Pakistan and evaluates its association with mortality, showing no significant difference in mortality across vitamin D status categories.	Not reported	Not reported	The study assessed vitamin D status only in neonates with sepsis, without a control group; vitamin D supplementation was administered as part of clinical management but was not evaluated as an intervention outcome.
31 [53]	Wang et al.	2022	China	Non-randomised experimental study	To investigate the prevalence of vitamin D insufficiency in hospitalized full-term neonates and to evaluate the factors associated with vitamin D insufficiency. and to explore the relation of vitamin D insufficiency with immunocompromise.	Yes (season of birth, maternal conditions, perinatal complications, and neonatal clinical variables)	471 neonates	Term neonates (≥37 weeks gestation)	Not specified	Not clearly defined	Serum 25(OH)D level	Neonatal venous blood	Postnatal- neonates	Immunoassay	Not applicable	Not applicable	Not applicable	Mean: 33.65±6.07	Deficiency: <20<br>Insufficiency: 20-30<br>Sufficiency: ≥30	Increased risk: The incidence rates of sepsis was higher in the vitamin D insufficiency group than those in the sufficiency group (p<0.05).	Sepsis incidence higher in vitamin D insufficiency group (5.26% vs 1.47%, p=0.044).	CD3+, CD4+ T cells; IgA	Supports growing evidence linking neonatal vitamin D insufficiency with infection risk; expands evidence from Asian neonatal populations; demonstrates association with immunological markers.	Maternal vitamin D levels not measured; no data on prenatal vitamin D supplementation; unable to assess maternal-neonatal vitamin D transfer	Need for maternal-neonatal vitamin D linkage studies; need for supplementation trials; need for standardized sepsis classification.	Study population limited to hospitalized neonates; may overrepresent sick infants; no differentiation between EOS and LOS; sepsis diagnostic criteria not clearly described.
32 [54]	Tofe-Valera et al.	2023	Spain	Case-control study	To monitor vitamin D and related biomarkers (PTH, calcium, phosphorus, MMP-8) in preterm infants at different time points and assess associations between vitamin D status and clinical outcomes, especially LOS.	Yes (weight; mechanical ventilation; parenteral nutrition duration; gestational age; gender; breastmilk feeding; days of hospitalization)	100 neonates (50 preterm; control group: 50 term)	≤32 weeks gestation and/or ≤1500 g birth weight	Late-onset (>72 h)	LOS is defined as the development of signs and symptoms of severe sepsis after 72h of life (blood culture, CBC, CRP, PCT).	Serum 25(OH)D level<br>Vitamin D supplementation	Cord blood; Neonatal venous blood; Maternal blood	Prenatal- maternal; Postnatal- neonates (at birth; 28 days of life; 4 months of life)	Immunoassay	1000 IU/day starting at 48h of life	Not reported	Enteral; Parenteral	Preterm infants:<br>Baseline: 17.97±9.35<br>28 days: 17.82±6.34<br>4 months: 33.95±13.4	Deficiency: <20	Increased risk: Regarding comorbidities among the preterm infants a relationship between 25(OH)D serum levels <20ng/ml and PTH levels >60pg/ mL and a higher incidence of LOS. At 28days of life, more than 90% of who developed sepsis showed vitamin D levels <20ng/ml.	Case-control design; incidence not reported.	PTH; MMP-8; osteocalcin; CRP	Suggests combined vitamin D-PTH status better predicts sepsis risk than vitamin D alone; supports longitudinal biomarker monitoring.	Single-center study; limited sample size; homogeneous population (Caucasian); results may not generalize	Lack of consensus on vitamin D thresholds in preterm infants; need for larger multicenter studies.	Study indicates vitamin D deficiency alone is insufficient predictor; endocrine-immune interaction may be key in neonatal infection risk assessment.
33 [55]	Zakerihamidi et al.	2023	Iran	Cross sectional study	To compare serum vitamin D levels in infected and uninfected preterm infants and investigate the relationship between vitamin D levels and neonatal infection/sepsis.	No	87 neonates (sepsis 42; control group: 45)	Gestational age <34 weeks or birth weight <2000g	Both	Infants with positive blood culture or cerebrospinal fluid and urine or clinical sepsis defined as two clinical signs accompanying (lethargy, apnea, respiratory distress, restlessness, seizures, need for mechanical ventilation, abdominal distention, hypotension and food intolerance) and two laboratory signs (20 000≤ WBC or ≤5000), PLT ≤150 000/µL and CRP≥10mg/dl without a positive culture.	Serum 25(OH)D level	Cord blood	Postnatal- neonates	Immunoassay	Not applicable	Not applicable	Not applicable	Control group: 23.31±9.40;<br>Sepsis group: 11.02±8.64	Severe deficiency: <10<br>Moderate deficiency: 10.1-20<br>Mild deficiency: 20.1-30<br>Normal: >30	Increased risk: The mean serum vitamin D levels in infected infants were lower than those in the uninfected cases, with rate 5.30±1.65 ng/ml in the clinical infection and 7.10±2.15ng/ml in the available cases along with sepsis plus meningitis.	48.3% of infants classified as infected (Early-onset sepsis: 35.7%; Late-onset sepsis: 64.3%)	CRP; WBC; PLT	Adds evidence from Middle East population; focus on preterm infants and cord blood vitamin D; supports growing literature linking vitamin D deficiency with neonatal sepsis risk.	Hospital-based control groups	Lack of randomized trials; limited mechanistic biomarkers.	Cross-sectional design limits causal inference; vitamin D measured only once (at birth); strong association but no risk estimates reported.
34 [56]	Toptan et al.	2023	Turkey	Cross sectional study	To investigate the association between blood vitamin D 25(OH)D levels and respiratory problems in very low birth weight infants.	No	66 neonates	Neonates 23–32 weeks gestation	Early-onset (<72 h)<br>Late-onset (after first 7 days)	Defined as a systemic condition originating from bacterial, viral or fungal causes and is associated with hemodynamic changes and clinical findings causing severe morbidity and mortality.	Serum 25(OH)D level	Neonatal venous blood	Postnatal- neonates (at birth (first 24 h); 28 days of life; 36 post-menstrual age)	LC-MS/MS	Not applicable	Not applicable	Not applicable	Day 1: median 4.95 (IQR 3.90-10.53)<br>Day 28/discharge: median 15.5 (IQR 7.5-37.8)	Deficiency: <20<br>Sufficiency: ≥20	Inconclusive: There was a significant correlation between 25(OH)D levels <20 ng/mL measured on the first day and sepsis (r=-0.557, p=0.003). However, there was no statistically significant correlation between 25(OH)D levels <20 ng/mL measured on the 28th day or at discharge and late sepsis (r=0.97, p=.094).	EOS was observed in 23 (34.85%) infants and LOS in 28 (42.42%) infants.	Not reported	Supports emerging evidence linking neonatal vitamin D deficiency with EOS and respiratory morbidity in VLBW infants.	Single-center study; small sample size; no term control group; maternal vitamin D levels not assessed	Need for larger, multicenter studies; need for maternal-neonatal vitamin D comparisons.	Study focused primarily on respiratory outcomes; sepsis analyzed as a secondary outcome; sepsis diagnostic criteria not explicitly defined.
35 [57]	Darnifayanti et al.	2024	Indonesia	Systematic review and meta-analysis	To elucidate the association of vitamin D receptor single nucleotide polymorphisms (FokI, TaqI, BsmI, BglI, and ApaI) with neonatal sepsis through qualitative and quantitative systematic review.	Yes (age, sex, BMI, delivery way, gestational age (reported in included studies quality assessment))	Total of 3 studies included. 500 neonates (250 sepsis cases; 250 healthy controls)	Term neonates (≥37 weeks gestation), aged <28 days; reported gestational age ranged from >37 weeks to 38.5±0.5 weeks	Not specified	Defined as organ dysfunction-related symptoms with blood culture-confirmed bacteremia in neonates <28 days old.	Serum 25(OH)D level	Neonatal venous blood; Maternal blood (one study)	Prenatal- maternal; Postnatal- neonates	Not reported	Not applicable	Not applicable	Not applicable	Not reported. The difference of vitamin D level between septic and healthy neonates was statistically significant (p <0.001).	Not reported	Protective: CC genotype and C allele of FokI; TT genotype of VDR TaqI variants had a protective effect against neonatal sepsis;	Case-control design; incidence not reported.	Serum 25(OH)D; VDR polymorphisms	Study synthesized emerging evidence linking VDR gene polymorphisms and neonatal sepsis susceptibility; demonstrated growing interest in genetic biomarkers and vitamin D-related immune regulation in neonatal sepsis.	Small number of included studies; high heterogeneity in pooled FokI analysis; BglI association reported in only one study; future studies with larger samples and multiple countries needed	Need for more robust evidence on VDR gene variants and neonatal sepsis; need for maternal gene polymorphism studies; need for additional studies evaluating BglI, ApaI, and other VDR polymorphisms.	Vitamin D supplementation was not evaluated; exact vitamin D assay methods and cut-off definitions were not consistently reported.; included studies focused mainly on term neonates with culture-confirmed sepsis.
36 [58]	Toaima et al.	2024	Egypt	Randomised controlled trial	To assess vitamin D status in full-term neonates with EOS and evaluate the effect of two different oral vitamin D supplementation regimens (400 IU/ day vs 800 IU/day) on recovery from sepsis.	No	66 neonates with EOS<br>(60 in recovery analysis; 6 deaths)	Term neonates (≥37 weeks gestation)	Early-onset (≤7 days of life)	Clinical and laboratory findings of EOS assessed using the Newborn Scale of Sepsis (score >10 considered septic), supported by CRP and blood culture where available.	Serum 25(OH)D level<br>Vitamin D supplementation	Neonatal venous blood	Postnatal- neonates (at enrolment: first day of sepsis; on recovery)	Immunoassay	Group A: 400 IU/day<br>Group B: 800 IU/day<br>Group C: No vitamin D supplementation (placebo)	From enrolment until recovery from sepsis	Oral	Mean±SD at enrolment: 18.12±3.6<br>Mean±SD on recovery: group A 22.08±6.3; group B 22.9±4.5; group C 18.95±1.9	Deficiency: <12<br>Insufficiency: 12-20<br>Sufficiency: >20	Protective	Study included only neonates diagnosed with sepsis; incidence not reported.	CRP	Contributes recent evidence on the therapeutic role of postnatal vitamin D supplementation in EOS, including dose-comparison between standard and higher supplementation regimens.	Maternal serum 25(OH)D levels not measured; seasonal variation and maternal clothing/sun exposure not assessed; small sample size	Optimal dosing of vitamin D in neonatal sepsis; long-term outcomes of supplementation; maternal-neonatal vitamin D interactions.	Strict inclusion of full-term neonates limits generalizability to preterm populations; clear RCT design with placebo group strengthens internal validity.
37 [59]	Obayed et al.	2024	India	Case-control study	To investigate the relationship between neonatal serum 25(OH) vitamin D levels and EOS in term neonates.	No	120 neonates (EOS: 60; control group: 60)	Term neonates (>37 weeks gestation)	Early-onset (≤72 h)	Defined as the presence of infection-related signs and symptoms, with or without the presence of bacteria in the bloodstream, occurring within the first 72h of life.	Serum 25(OH)D level	Neonatal venous blood	Postnatal- neonates (≤72 hours of life)	Not reported	Not applicable	Not applicable	Not applicable	Mean±SD: 21.4±10.2<br>Range: 8.1-53.3	Not reported	Increased risk: Lower vitamin D levels observed in EOS cases compared with controls.	Case-control design; incidence not reported.	CRP; TLC; PLT	Supports accumulating evidence linking low neonatal vitamin D levels with EOS.	Not reported	Limited data on vitamin D and EOS in neonates; need for larger, multicentre and mechanistic studies.	Only term neonates included, limited generalisability to preterm population; method of vitamin D assay not reported; maternal vitamin D mentioned in aim but not fully analysed in results.
38 [60]	Mohamed et al.	2024	Egypt	Case-control study	To evaluate the association between vitamin D levels and the risk of EOS in full-term neonates.	Yes (Apgar score at 1min, vitamin D status, respiratory difficulties, mottling)	90 neonates (EOS: 45; control group: 45)	Term neonates (≥37 weeks gestation)	Early-onset (≤72 h)	EOS defined by clinical signs and laboratory criteria by Gitto et al. [23] within the first 72 hours of life, including CRP, CBC, and blood culture results, categorized as highly probable, probable, possible, or no sepsis.	Serum 25(OH)D level	Neonatal venous blood	Postnatal- neonates (≤72 hours of life)	Immunoassay	Not applicable	Not applicable	Not applicable	Sepsis group: median 15.4 (6.1-57.2)<br>Control group: median 30.4 (5.1-107)	Deficiency: <20<br>Insufficiency: 20-30<br>Sufficiency: >30	Increased risk: Low serum vitamin D levels associated with higher risk of EOS; longer NICU stay with deficient vitamin D levels.	Case-control design; incidence not reported.	CRP; WBC; TLC	Supports accumulating evidence linking low neonatal vitamin D levels with increased risk of EOS in term neonates.	Absence of maternal serum vitamin D measurements; small sample size; lack of post-supplementation vitamin D assessment in septic neonates	Need for studies assessing maternal-neonatal vitamin D status and interventional studies evaluating vitamin D supplementation effects on neonatal sepsis outcomes.	Study restricted to full-term neonates; findings may not be generalizable to preterm populations; sepsis definition included clinical and laboratory criteria rather than culture-proven sepsis alone.
39 [61]	Dua et al.	2024	India	Case-control study	To evaluate and compare serum vitamin D levels in term neonates with sepsis and without sepsis.	No	60 neonates (sepsis: 30; control group: 30)	Term neonates (≥37 weeks gestation)	Early-onset (≤72 h)<br>Late-onset (>72 h)	EOS defined by clinical signs and laboratory criteria by Gitto et al. [23] within the first 72 hours of life, including CRP, CBC, and blood culture results, categorized as highly probable, probable, possible, or no sepsis.	Serum 25(OH)D level	Neonatal venous blood	Postnatal- neonates (at time of sepsis diagnosis)	Immunoassay	Not applicable	Not applicable	Not applicable	Sepsis group: mean 16.0±10.5<br>Control group: mean 29.07±8.4	Deficiency: <12<br>Insufficiency: 12-20<br>Sufficiency: >20-50	Increased risk: Serum vitamin D levels were significantly lower in septic neonates compared to healthy controls, suggesting a potential role of vitamin D deficiency in the pathogenesis of sepsis.	Case-control design; incidence not reported.	CRP; WBC; ANC; PLT	Adds evidence supporting the association between neonatal vitamin D deficiency and increased risk and severity of sepsis, particularly EOS.	Small sample size; Single-center study; Lack of assessment of maternal vitamin D status; Potential unmeasured confounders (environmental and maternal factors)	Need for larger multicenter studies; need to evaluate maternal-neonatal vitamin D relationship and supplementation effects.	Study includes only term neonates; no vitamin D supplementation intervention evaluated; observational design limits causal inference.
40 [62]	Sharma et al.	2024	India	Cross sectional study	To explore the relationship between vitamin D level and the incidence of LOS in term neonates.	No	90 neonates (sepsis group: 45; control group: 45; mothers included	Term neonates (≥37 weeks gestation)	Late-onset (>72 h)	Blood culture-proven sepsis diagnosed between 72 hours and 28 days of life.	Serum 25(OH)D level	Neonatal venous blood; Maternal blood	Prenatal- maternal; Postnatal- neonates (between 72 hours and 28 days of life)	Immunoassay	Not applicable	Not applicable	Not applicable	Neonates: sepsis group: mean 20.40±7.32;<br>control group: mean 25.55±4.11;<br>Mothers: sepsis group: mean 21.63±10.16;<br>control group: mean 28.89±4.60	Deficiency: <20<br>Insufficiency: 20-29<br>Sufficiency: ≥30	Increased risk: There were statistically significant differences in the mean vitamin D levels (p=.0001) between the neonates in the sepsis and control groups as well as in the mean vitamin D levels between the mothers of the neonates with sepsis and control group neonates.	Study included only neonates diagnosed with sepsis; incidence not reported.	Not reported	Provides recent evidence supporting an association between neonatal and maternal vitamin D deficiency and LOS in term neonates.	Small sample size; dose and duration of maternal vitamin D supplementation not considered	Need for larger studies and evaluation of maternal vitamin D supplementation effects.	Only term neonates were included; findings cannot be generalized to preterm populations; observational cross-sectional design limits causal inference.
41 [63]	Ergon et al.	2024	Turkey	Cross sectional study	To evaluate whether standard-dose vitamin D supplementation provides adequate serum 25(OH)D levels in exclusively breastfed term neonates born with different cord blood vitamin D levels and to assess associated neonatal morbidities.	No	86 neonates	Term neonates (≥37 weeks gestation)	Both	Sepsis classified as clinical sepsis or culture-proven sepsis.	Serum 25(OH)D level<br>Vitamin D supplementation	Cord blood; Neonatal venous blood	Postnatal- neonates (at birth; first month of life)	Immunoassay	400 IU/day	First month of life	Oral	Cord blood: deficient group: 7±1.7;<br>insufficient group: 14±1.4; optimal group: 23±3.5<br>After first month: deficient group: 15±4.2;<br>insufficient group: 20±3.6; optimal group: 27±4.2	Deficiency: <12<br>Insufficiency: 12-19<br>Sufficiency: ≥20	Increased risk: Throughout the first month of life, infants in the deficiency group had more EOS than newborns in the other groups (p = 0.05).	Incidence not reported.	Not reported	Adds evidence linking low cord blood vitamin D levels with increased neonatal morbidity despite standard supplementation, supporting individualized supplementation approaches.	Single-center study; small sample size; short follow-up period; lack of long-term neonatal outcomes; no direct maternal serum vitamin D measurements	Optimal vitamin D dosing strategies in neonates; long-term outcomes of neonatal vitamin D deficiency; relationship between maternal vitamin D levels and neonatal outcomes.	Exclusively term neonates included; standardized supplementation applied to all infants; strong correlation between cord and first-month serum 25(OH)D levels; findings support reconsideration of uniform vitamin D prophylaxis dosing.
42 [64]	Delair et al.	2024	Nigeria	Cohort study	To assess the association between maternal and cord blood vitamin D metabolites (25(OH)D, 3-epi-25(OH)D3, 24,25(OH)2D3) and newborn sepsis evaluation, as well as maternal risk factors and newborn outcomes, in Nigerian mother-infant dyads.	Yes (maternal age)	Neonates: 536; Mothers: 534	Inclusion criteria: gestational age ≥24 weeks; mean gestational age: 38.4±2.2 weeks	Early-onset (≤72 h)<br>Late-onset (>72 h, within first 28 days of life)	Defined as bloodstream infection in first 28 days of life; evaluation included screening for bacteremia and meningitis as clinically indicated.	Serum 25(OH)D;<br>3-epi-25(OH)D3;<br>24,25(OH)2D3 levels	Cord blood; Maternal blood	Prenatal- maternal; Postnatal- neonates (at birth)	LC-MS/MS	Not applicable	Not applicable	Not applicable	Maternal mean: 36.72±11.6<br>Cord-blood mean: 24.52±8.14	Deficiency: <20<br>Insufficiency: 20-29<br>Sufficiency: ≥30	Increased risk: Maternal and infant median levels of 25(OH)D, 24,25(OH)2D3, and 3-epi-25(OH)D3 are not significantly associated with newborn sepsis evaluation. The median values of percent 3-epi-25(OH)D3 are higher in the neonates who were evaluated for sepsis than in those who were not, and this difference is statistically significant (6.18 vs. 5.68, p=0.036).	34 of 526 newborns (6.3%) evaluated for sepsis.	Serum 25(OH)D; 3-epi-25(OH)D3; 24,25(OH)2D3	First study in a Nigerian population to assess vitamin D metabolites (including epimers) and their association with newborn sepsis evaluation using LC-MS/MS methodology.	Study was not powered to detect an association between neonatal sepsis and 25(OH)D deficiency; lacked detailed data on dietary intake and vitamin D supplementation of mothers	Lack of studies on vitamin D epimers and neonatal sepsis; limited data on vitamin D metabolites in African mother-infant dyads; unclear biological role of 3-epi-25(OH)D3 in neonatal immunity.	Strong methodological strength: use of LC-MS/MS allowing separation of epimers; findings highlight importance of reporting vitamin D metabolites beyond total 25(OH)D.
43 [65]	Kaveh et al.	2025	Iran	Case-control study	To investigate the relationship between maternal and neonatal serum vitamin D levels in term infants with and without neonatal sepsis.	Yes (infant age, sex, maternal age, maternal education)	320 participants (160: 80mothers-80infant pairs with sepsis; 160 control mother-infant pairs)	Term neonates (≥37 weeks gestation)	Both	Presence of at least two clinical and laboratory manifestations of sepsis.	Serum 25(OH)D level	Neonatal venous blood; Maternal blood	Prenatal- maternal; Postnatal- neonates (>72 hours of life)	Not reported	Not applicable	Not applicable	Not applicable	Neonates: median 18.1 (12.4-24.1) in sepsis group; 21.2 (14.8-31) in control group;<br>Mothers: median 23.8 (15.0-30.0) in sepsis group; 28.6 (17.1-34.0) in control group	Deficiency: <10<br>Moderate deficiency: 10-20<br>Mild deficiency: 20-30<br>Normal: 30-100	Increased risk: Low maternal vitamin D associated with higher neonatal sepsis risk.	Case-control design; incidence not reported.	Not reported	Adds recent evidence supporting maternal vitamin D deficiency as an independent risk factor for neonatal sepsis in term infants; reinforces maternal-neonatal vitamin D correlation.	Not reported	Need for prospective interventional studies and randomized trials evaluating maternal vitamin D supplementation and neonatal sepsis outcomes.	Strong maternal-neonatal vitamin D correlation; neonatal vitamin D levels alone were not significantly associated with sepsis, highlighting maternal status as more influential.
44 [66]	Rabbany et al.	2025	Bangladesh	Cross sectional study	To determine the association between serum 25(OH) vitamin D deficiency and LOS in term and late preterm neonates.	No	178 neonates (LOS: 89; control group: 89)	Preterm included (34-36+6 weeks); term (≥37 weeks)	Late-onset (>72 h)	LOS was defined as the development of signs and symptoms of severe sepsis after 72h of life and a positive sepsis screen eg. ≥2 abnormal parameters among CBC, I/T, CRP and blood or cerebrospinal culture.	Serum 25(OH)D level	Neonatal venous blood; Maternal blood	Postnatal- neonates (>72 hours of life)	Immunoassay	Not applicable (supplemented neonates excluded)	Not applicable	Not applicable	Sepsis group: 13.99±6.07;<br>Control group: 20.56±5.93	Deficiency: <20<br>Insufficiency: <29<br>Sufficiency: 30-50	Increased risk: The mean serum 25(OH) vitamin D level in the sepsis case group was significantly lower than in controls (p<0.001). Significant positive correlation of neonatal 25(OH)D with maternal in both case (r=0.983; p<0.001) and control (r=0.970; p<0.001).	Study compared LOS cases vs non-septic controls.	Not reported	Adds evidence from Bangladesh, where data were previously scarce; supports growing literature linking vitamin D deficiency and neonatal sepsis; reinforces maternal-neonatal vitamin D correlation.	Not reported	Lack of national data in Bangladesh on vitamin D and neonatal sepsis.	Strong maternal-neonatal vitamin D correlation suggests maternal supplementation strategies may be important; exclusion of previously hospitalized or supplemented infants improves internal validity.
45 [67]	Verisokina et al.	2025	Russian Federation	Non-randomised experimental study	To enhance the effectiveness of prevention and correction of hypovitaminosis D in preterm infants and to assess the effect of cholecalciferol supplementation on serum 25(OH)D, pro-inflammatory cytokines (IL-1β, IL-6, TNF-α), and cathelicidin levels.	No	57 neonates (intervention group: 33; control group: 24)	Median gestational age (weeks): intervention group: 30.0 [28.0; 32.0]; control group: 31.0 [29.0; 33.0]	Not specified	Not clearly defined	Serum 25(OH)D level<br>Vitamin D supplementation	Neonatal venous blood	Postnatal- neonates (at birth: within first 24h; after 21 days of life)	Immunoassay	1000 IU/day	21 days	Oral	Before supplementation: median 18.9 [11.7; 21.9];<br>After 21 days: median 33.8 [19.8; 46.9]	Severe deficiency: <10<br>Deficiency: 10-20<br>Insufficiency: 20-30<br>Sufficiency: >30	Not assessed	Not reported.	IL-1β; IL-6; TNF-α; cathelicidin	Provides recent evidence (2025) supporting the immunomodulatory effects and safety of early postnatal cholecalciferol supplementation (1000 IU/day) in preterm infants.	Need for further clinical and molecular biological studies to better understand vitamin D mechanisms	Lack of unified clinical guidelines in the Russian Federation for vitamin D supplementation in preterm infants.	Study focuses on immunological biomarkers rather than clinical sepsis outcomes; non-randomised design and absence of explicit sepsis diagnostic criteria limit applicability to sepsis risk assessment.

The following abbreviations are used in the table: LOS- late-onset sepsis; EOS- early-onset sepsis; PROM- premature rupture of membranes; LC-MS/MS- liquid chromatography-tandem mass spectrometry; CRP- C-reactive protein; PCT- procalcitonin; PLT- platelet count; WBC- white blood cell count; CBC- complete blood count; I/T- immature-to-total neutrophil ratio; TLC- Total leukocyte count; ANC- absolute neutrophil count; GLR- granulocyte-to-leukocyte ratio; LL-37- Cathelicidin; VDR- Vitamin D receptors; RR- relative risk; CI- 95% confidence interval
